# Federated Gastrointestinal Lesion Classification with Clinical-Entropy Guided Quantum-Inspired Token Pruning in Vision Transformers

**DOI:** 10.3390/diagnostics16071027

**Published:** 2026-03-29

**Authors:** Muhammad Awais, Ali Mustafa Qamar, Umair Khalid, Rehan Ullah Khan

**Affiliations:** 1 Department of Computer Science, College of Computer, Qassim University, Buraydah 52571, Saudi Arabia; mu.aslam@qu.edu.sa; 2 Build My Dapp Pvt. Ltd., Islamabad 44000, Pakistan; 3 Department of Information Technology, College of Computer, Qassim University, Buraydah 52571, Saudi Arabia; re.khan@qu.edu.sa

**Keywords:** federated learning, gastrointestinal disorders, optimization, evolutionary computation, deep learning

## Abstract

**Background:** Gastrointestinal (GI) cancers remain a major global health concern, where timely and accurate interpretation of endoscopic findings plays a decisive role in patient outcomes. In recent years, deep learning–based decision support systems have shown considerable potential in assisting GI diagnosis; however, their broader adoption is often limited by patient privacy regulations, uneven data availability, and the fragmented nature of clinical data across institutions. Federated learning (FL) offers a practical solution by enabling collaborative model training while keeping patient data local to each hospital. **Methods:** Vision Transformers (ViTs) are particularly well suited for endoscopic image analysis due to their ability to capture long-range contextual information. Nevertheless, their high computational and communication costs pose a significant challenge in federated settings, especially when data distributions vary across clients. To address this issue, we propose a privacy-preserving federated framework that combines ViTs with a Clinical-Entropy Guided Quantum Evolutionary Algorithm (CEQEA) for adaptive token pruning. The CEQEA leverages the diagnostic diversity of each client’s local dataset to guide population initialization, evolutionary updates, and mutation strength, allowing the pruning strategy to adapt naturally to different clinical profiles. **Results:** The proposed framework was evaluated on curated upper- and lower-GI tract subsets of the HyperKVASIR dataset under realistic non-IID federated conditions. On the final test sets, the model achieved a mean micro-averaged accuracy of 92.33% for lower-GI classification and 90.19% for upper-GI classification, while maintaining high specificity across all diagnostic classes. At the same time, the adaptive pruning strategy reduced the number of tokens processed by approximately 40% and decreased the number of required federated communication rounds by 33% compared to ViT-based federated baselines. **Conclusions:** Overall, these results indicate that entropy-aware, quantum-inspired evolutionary optimization can effectively balance diagnostic performance and efficiency, making transformer-based models more practical for privacy-preserving, multi-institutional gastrointestinal endoscopy.

## 1. Introduction

Gastrointestinal (GI) disorders represent a major global health concern and contribute substantially to worldwide morbidity and mortality [1]. These conditions span a broad clinical spectrum, ranging from benign inflammatory diseases to pre-malignant abnormalities such as adenomatous colon polyps, and ultimately to malignant cancers, including colorectal, gastric, and esophageal carcinoma. Among these, GI malignancies remain particularly critical, accounting for nearly one-third of global cancer-related deaths, with more than 3.7 million fatalities reported in recent global assessments [2]. In this study, the term GI disorders refers to the overall disease spectrum affecting the gastrointestinal tract, whereas GI lesions denote localized abnormalities visible during endoscopic examination that may correspond to benign, pre-malignant, or malignant conditions. Early and accurate identification of such lesions is therefore essential for timely therapeutic intervention and improved patient outcomes. In clinical practice, diagnosis primarily relies on endoscopic examination supported by imaging techniques such as narrow-band imaging and chromoendoscopy. However, interpretation of endoscopic findings is highly dependent on clinician expertise, and subtle visual differences between lesion types can lead to inter-observer variability and missed detections. These challenges motivate the development of reliable computer-aided systems to support consistent and accurate gastrointestinal diagnosis.

Artificial intelligence (AI) techniques have increasingly been explored to assist automated analysis of endoscopic imagery. Deep learning approaches, particularly convolutional neural networks (CNNs), have demonstrated promising performance in gastrointestinal lesion detection and classification tasks due to their ability to learn hierarchical visual representations from large image datasets [2,3]. Nevertheless, CNN-based architectures primarily rely on localized feature extraction and may struggle to capture long-range contextual relationships within complex endoscopic scenes. Vision Transformers (ViTs) have recently emerged as a powerful alternative by representing images as sequences of patches and modeling global dependencies through self-attention mechanisms [4]. Despite these advantages, robust transformer-based medical imaging systems typically require large and diverse datasets, which are difficult to obtain because clinical data are distributed across institutions and protected by strict privacy regulations.

Federated learning (FL) provides a promising framework to address this limitation by enabling collaborative model training across multiple healthcare institutions without sharing raw patient data. However, integrating transformer architectures within federated environments introduces additional efficiency challenges. The quadratic computational complexity of the self-attention mechanism increases memory usage and training costs on resource-constrained clinical devices, while the transmission of large model updates may create substantial communication overhead during federated optimization [5]. Moreover, clinical datasets across institutions are typically heterogeneous and exhibit non-IID distributions, reflecting differences in patient populations, diagnostic priorities, and clinical workflows. In practical deployments, the clinical conditions described above are represented as predefined endoscopic classes in curated datasets, corresponding to specific gastrointestinal lesion categories used for model training and evaluation. Consequently, efficiency strategies designed for centralized learning may not effectively adapt to the varying clinical distributions encountered across federated clients.

Motivated by these challenges, this study proposes a Clinical-Entropy Guided Quantum Evolutionary Algorithm (CEQEA) for adaptive token pruning in federated Vision Transformers applied to gastrointestinal endoscopic image classification. The proposed framework dynamically adjusts token pruning strategies according to the entropy and diagnostic composition of each client’s local dataset, enabling client-specific adaptation within heterogeneous federated environments. By integrating quantum-inspired evolutionary optimization with federated transformer training, the approach aims to improve computational and communication efficiency while preserving diagnostically relevant visual information. The method is evaluated on curated endoscopic datasets representing clinically meaningful GI lesion classes under realistic, non-IID federated learning conditions to access its effectiveness for scalable, privacy-preserving gastrointestinal image analysis.

## 2. Literature Review

The existing literature on deep learning for gastrointestinal endoscopy can be broadly categorized into three interconnected paradigms: (i) deep CNN–based GI lesion classification methods, primarily trained on benchmark datasets such as Kvasir; (ii) approaches that employ Vision Transformers for centralized endoscopic image analysis; and (iii) frameworks based on utilizing ViTs with FL settings, along with efficiency-enhancing strategies to address clinical and computational constraints.

### 2.1. CNN-Based Methods for GI Lesion Classification

The initial studies focused on using transfer learning on pre-trained CNNs for multiclass classification of GI lesion images. In a seminal work [6], the authors presented the Hyper KVASIR dataset, which later became the benchmark for AI-assisted GI endoscopy research. In their study, the authors evaluated the performance of several pretrained CNN architectures on the KVASIR dataset, including ResNet-50, ResNet-152, DenseNet-161, as well as ensemble models combining ResNet-152 and DenseNet-161. Their experimental results reported classification accuracies ranging from 87% to 96%. Siddiqui et al. [3] proposed SNeT, an 80-layer custom CNN model explicitly designed for GI analysis. The custom CNN employs 26 convolutional layers with residual connections to learn hierarchical features specific to GI images. Further, the authors incorporated a Minimum Redundancy Maximum Relevance (MRMR) feature selection algorithm to enhance model efficiency. The model training and evaluation were carried out in a cross-dataset manner, yielding accuracies of 98.45% on the KVASIR v1 dataset and 97.83% on the KVASIR v2 dataset. Khan et al. [7] presented a two-stage pipeline for GI disease classification. The first stage comprises a saliency module that extracts regions of interest (ROIs) likely to contain pathological findings. Next, pre-trained DenseNet-201 is employed as a feature extractor. The features are then refined using a modified moth-flame optimization algorithm. The framework achieved accuracies of 98% on KVASIR and 99.6% on a private dataset. Similarly, Jha et al. [8] proposed GastroVision, a multi-center open-access GI endoscopy dataset that includes different anatomical landmarks, pathological abnormalities, polyp removal cases and normal findings. The experimental evaluation was performed using multiple deep CNNs, including ResNet-50, ResNet-152, EfficientNet-B0, DenseNet-121 and DenseNet-169 DenseNet-121 achieved the best performance metrics. Sedighipour [9] proposed an autoencoder along with an Xception model. The autoencoder was first trained on thousands of unlabeled data images; the encoder was then used to extract features. The Xception model was also adopted as a feature extractor. The combined feature set was later classified using CNN. An evaluation on the KVASIR dataset reported a 5% performance gain using the ensemble base approach compared to some existing methods.

### 2.2. ViT-Based Methods in GI Endoscopy

The application of ViTs in GI endoscopy has been explored in several recent studies. Chen et al. [10] first self-collected a dataset of endoscopic images belonging to chronic atrophic and chronic nonatrophic gastritis (CAG) classes. The images were then used to train the Swin Transformer and SSFormer models to detect anatomical structures of the stomach, CAG, and atrophic lesion regions. The Swin transformer obtained an accuracy of 98% in recognizing anatomical structures of the stomach. Furthermore, the SSFormer demonstrated accuracy comparable to that of an endoscopist in segmenting lesions. Ayan [11] presented a comparative analysis of CNN and ViT-based models for GI disease classification. The work utilized thirteen different CNNs along with two ViTs. The study concluded that although ViTs often outperform CNN baselines on GI images, properly tuned and optimized CNNs perform better with limited or imbalanced datasets. In addition to utilizing CNNs and ViTs alone for classification tasks, few studies have also investigated a combination of both models to extract a richer set of both local and global features. For example, the work by Tanwar et al. [12] proposed a hybrid framework utilizing EfficientNetB0 in combination with ViT. The EfficientNetB0 was utilized to extract local textures and multi-scale features corresponding to changes in the GI tract. Further, the ViT encoder was employed to detect subtle disease patterns and precursors of disease diffusion. The work demonstrated a high accuracy of up to 99% on the Kaggle dataset. Similarly, in [13], the authors present a multi-task network named TransMT-Net, which integrates ViT encoders with segmentation and active learning for improving both lesion localization and classification accuracy in endoscopic imagery. Model performance was evaluated on a custom dataset derived from the CVC-ClinicDB dataset. The model achieved 96.94% classification accuracy and 77.76% Dice Similarity Coefficient in segmentation tasks. These works highlight the emerging potential of ViTs in improving GI image classification tasks, along with highlighting issues related to data imbalance, model generalization, and modality variations. Recent advances in GI disease classification have also explored multimodal approaches combining advanced imaging with AI. Chou et al. [14] demonstrated integrating hyperspectral imaging with deep learning to enhance the classification of selected gastrointestinal diseases, achieving improved diagnostic accuracy by leveraging spectral information beyond conventional RGB endoscopy.

### 2.3. ViT-FL Based Methods

Integration of ViTs in federated learning has recently attracted research interest, with the need to learn powerful global representations from heterogeneous, multi-center endoscopic data while preserving patient privacy. Alkhunaizi et al. [15] investigated several strategies for fine-tuning ViT parameters, such as visual prompt tuning, low-rank decomposition of visual prompts, stochastic block attention fine-tuning, and hybrid methods like low-rank adaptation. The purpose of these strategies was to achieve a good trade-off between efficiency and accuracy in non-IID data, a common challenge in FL. Darzi et al. [16] proposed a method that leverages the multi-head attention mechanism in ViTs to align the representations of heterogeneous data across clients. Their work, evaluated on a lung cancer dataset across various levels of heterogeneity, demonstrated competitive performance against baselines. Similarly, Kirchner et al. [17] introduced federated EndoViT, a privacy-preserving technique for training surgical foundation models using FL on endoscopic image data. The authors adapted the EndoViT Vision Transformer to a federated setting, combining Adaptive Federated Sharpness-Aware Minimization (FedSAM) and Stochastic Weight Averaging (SWA) to accommodate data heterogeneity across institutions. After pretraining a masked autoencoder cooperatively among nine clients without data sharing, the model is fine-tuned on three downstream tasks: surgical scene segmentation (SSS), action triplet recognition (ATR), and surgical phase recognition (SPR). Their results demonstrate that FL-EndoViT, when fully fine-tuned, achieves performance comparable to or even superior to its centralized counterpart, particularly in SSS with high-resolution images and in ATR with full datasets. The application of ViTs in medical imaging has extended beyond GI endoscopy to other clinical domains. Wang et al. [18] demonstrated the efficacy of ViT architectures in glaucoma detection through a novel hyperspectral imaging band selection approach, achieving superior performance by integrating spectral band optimization with transformer-based feature extraction.

To enhance the efficiency of ViTs, several recent works have investigated token pruning strategies. Rao et al. [19] introduced DynamicViT, which combines a lightweight MLP-based prediction network that assigns a score to each token’s relevance and dynamically prunes less informative tokens in ViT during inference. Liu et al. [20] created Efficient-VIT, which operates on a token rearrangement and fusion paradigm, where related patch tokens are merged in later transformer layers to reduce computational burden. In [21], an attention-based token pruning technique is suggested that eliminates tokens with low attention scores relative to class tokens utilizing the self-attention mechanism itself as a pruning criterion.

A critical examination of the literature reveals a significant gap in federated medical imaging: while centralized token pruning methods—such as the static, globally optimized policies and rigid attention-based thresholds—improve efficiency, they lack a client-adaptive, privacy-preserving mechanism suitable for ViTs in non-IID federated environments. For example, pruning tokens with the lowest attention scores optimized on one client’s data may eliminate tokens crucial for diagnosis on another client’s data. As emphasized by Li et al. [22], heterogeneity is the rule, not the exception, in FL. The algorithms must be robust to address this. There is a lack of research on client-specific, adaptive pruning strategies that can tailor model efficiency to local data characteristics within a federated ViT framework for GI disease classification.

## 3. Materials and Methods

In clinical gastroenterology, the diagnosis of gastrointestinal (GI) disorders relies primarily on endoscopic visualization of the digestive tract, where physicians identify abnormal tissue patterns, lesions, or anatomical landmarks. In this study, the input to the proposed framework consists of gastrointestinal endoscopic images obtained during routine endoscopic procedures and curated from publicly available datasets. Each image represents a visual observation of the GI tract and is annotated according to predefined endoscopic classes corresponding to clinically relevant categories, such as benign lesions, pre-malignant polyps, malignant lesions, and anatomical landmarks. Accordingly, the learning task addressed in this work is a multi-class image classification problem in which the model predicts the endoscopic class associated with each image, thereby performing a clinically recognizable task of gastrointestinal lesion category classification. To support collaborative model development across institutions while preserving patient privacy, the classification model is trained within a federated learning framework, where multiple clients train locally on their own endoscopic data and share only model updates. To improve the feasibility of transformer-based models under such distributed clinical constraints, the proposed framework integrates a Clinical-Entropy Guided Quantum Evolutionary Algorithm (CEQEA) for adaptive token pruning in Vision Transformers, enabling efficient training while maintaining diagnostic classification performance across heterogeneous clinical datasets.

### 3.1. DataSets

The experimental evaluation in this study utilizes gastrointestinal endoscopic images derived from the HyperKVASIR dataset, a large publicly available collection of images obtained during routine endoscopic procedures. The dataset includes images acquired from both upper gastrointestinal endoscopy (gastroscopy) and lower gastrointestinal endoscopy (colonoscopy), covering multiple organs of the digestive tract, including the esophagus, stomach, and colon. These images represent a wide range of clinically relevant findings encountered during routine endoscopic practice, including benign lesions, pre-malignant polyps, inflammatory conditions, malignant tumors, and normal anatomical structures. In addition, the dataset contains anatomical landmarks and procedural views commonly used for orientation during endoscopic examinations.

Within the experimental framework, these clinical findings are represented as predefined endoscopic classes, allowing the classification task to directly correspond to clinically interpretable categories of gastrointestinal lesions and anatomical structures. The images originate from real clinical examinations and reflect the variability encountered in routine practice, including differences in illumination, tissue appearance, viewing angles, and endoscopic equipment. Images with severe artifacts or insufficient visual quality are typically excluded during dataset curation to ensure reliable annotation and evaluation. Consequently, the dataset provides a diverse and clinically relevant benchmark for developing and evaluating automated systems for gastrointestinal lesion classification.

**Figure 1:**
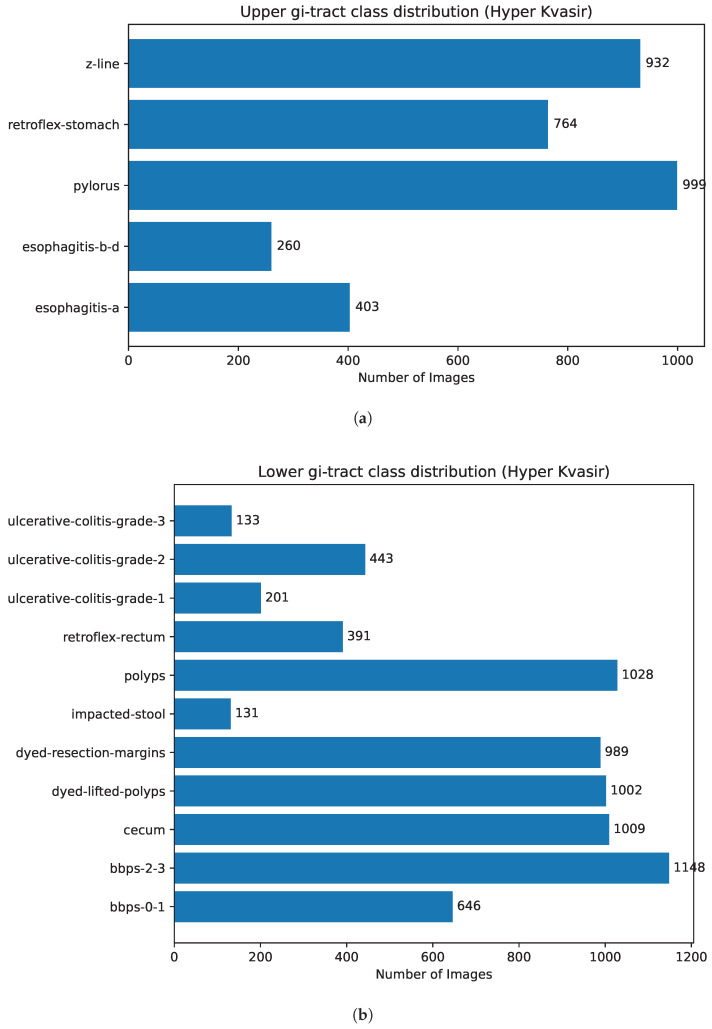
Class-wise distribution of the curated HyperKVASIR dataset showing (**a**) upper Gastrointestinal (GI) tract categories and (**b**) lower GI tract categories.

**Figure 2:**
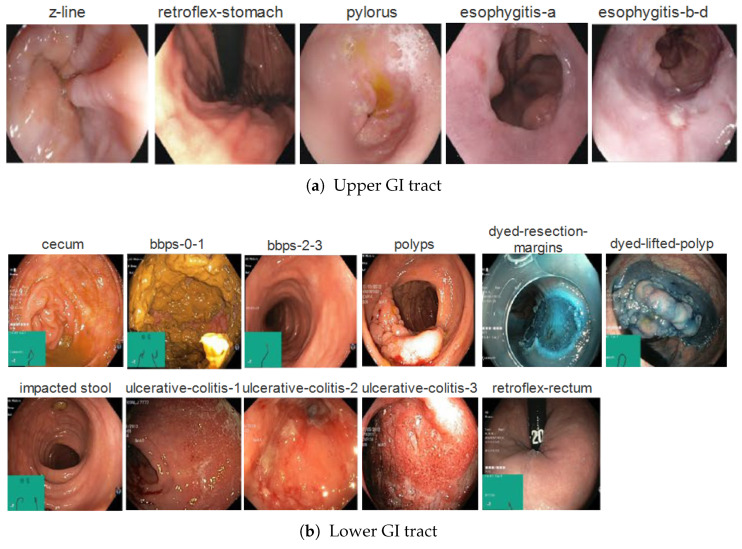
Sample images of the upper and lower GI tract in the curated HyperKVASIR dataset.

The HyperKVASIR dataset, introduced by Borgli et al. [6] at the Simula Research Laboratory in Norway, originally contains 110,079 images and 374 videos representing a broad spectrum of gastrointestinal findings across the upper and lower GI tract. The dataset includes both labeled and unlabeled data, enabling research in supervised, semi-supervised, and self-supervised learning scenarios. In this work, a curated subset of the HyperKVASIR dataset is used, consisting of 10,479 annotated endoscopic images distributed across 16 diagnostic classes, covering both upper and lower gastrointestinal tract findings. Figure 1 illustrates the class-wise distribution of images across both subsets, while Figure 2 presents representative endoscopic images from all classes.

The upper GI tract dataset is composed of a total of 3358 images, belonging to 5 classes representing inflammatory conditions and landmarks of esophageal and gastric pathology. These classes include esophagitis-a, esophagitis-b-d, pylorus, retroflex-stomach, and z-line. The pylorus, which represents the stomach outlet and is a significant anatomical landmark, is the most common class (999 images). The z-line (932 images), which represents the gastroesophageal junction and whose appearance is crucial for Barrett’s esophagus surveillance. According to the Los Angeles classification system, esophagitis-a (403 images) and esophagitis-b-d (260 images) represent the range of reflux-related mucosal injury. Furthermore, complete gastric examination techniques necessary for a comprehensive upper endoscopy are documented by retroflex-stomach views (764 images).

The lower GI tract subset comprises 7,121 images across 11 classes, representing colonic and rectal findings encountered in clinical practice. The most dominant class is bbps-2-3, comprising 1148 images. It reflects colonoscopy cases with fair to good bowel preparation, where the colonic mucosa is sufficiently clean for clinical assessment. This category is very important for representing everyday clinical observations and plays a vital role in providing valuable context for learning robust features for downstream lesion detection and classification tasks. Polyps is another significant class (1028 images), representing colorectal polyps, abnormal tissue growths on the inner lining of the colon or rectum. The cecum class (1009 images) contains images of the cecum, the first part of the large intestine, where the small intestine meets the colon. Visualizing the cecum images is very important, as it indicates that the entire colon has been inspected during colonoscopy. The dyed-lifted-polyps (1002 images) and dyed-resection-margins (989 images) classes represent the key stages during the endoscopic mucosal resection procedure. The former depicts the polyp removal stage, in which a dye-containing solution is injected beneath the lesion to lift it for easier resection. The latter class contains images after resection showing mucosal margins to assess completeness of removal. The ulcerative colitis class shows images of colon inflammation at three levels (grades) of severity. Grade 1 (201 images) represents mild inflammation with minor mucosal changes; grade 2 (443 images) shows more prominent signs such as redness and increased sensitivity of the intestinal lining; and grade 3 (133 images) represents severe inflammation and sometimes bleeding.

### 3.2. Pipeline of Proposed Framework

**Figure 3:**
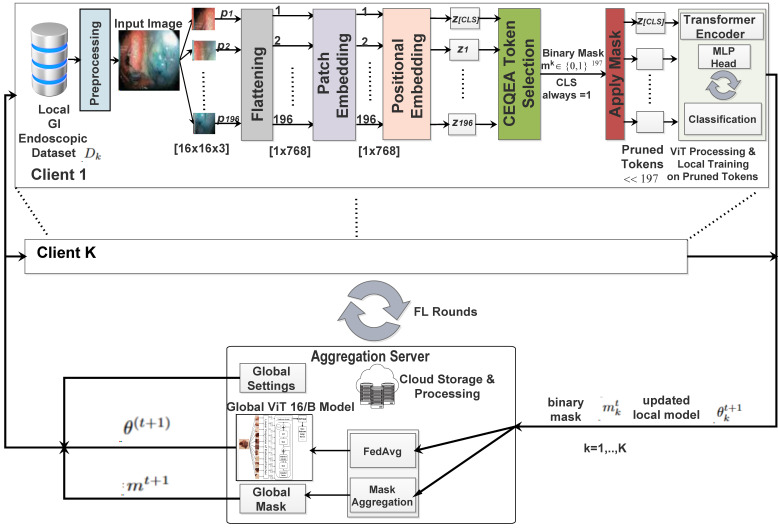
Pipeline of Proposed Framework for Federated GI Lesion Classification Utilizing Clinical-Entropy Guided Quantum Evolutionary Algorithm (CEQEA)-based token pruning for Vision Transformers (ViTs).

Figure 3 demonstrates the end-to-end workflow of the proposed framework for federated GI lesion classification utilizing CEQEA-based token selection for ViTs. The pipeline integrates a ViT-16/B as a backbone global model for feature learning and classification, along with CEQEA for adaptive token pruning. The proposed methodology minimizes client-side ViT complexity and federated communication cost by dynamically pruning superfluous patch-level tokens, while maintaining model generality and enabling privacy-preserving distributed training across several institutions.

The FL framework consists of a central aggregation server and *K* clients, and operates over *R* federated communication rounds. During each round;

- The global ViT-16/B model is broadcast from the aggregation server to each participating client.
- Each participating client preprocesses its local endoscopic images (from the IID-distributed HyperKVASIR subsets), resizing and augmenting as required.
- The preprocessed images undergo tokenization and patch embedding using ViT-16/B, generating a sequence of token representations alongside a global [CLS] token.
- The CEQEA module is operated locally to develop a client-specific binary pruning mask, optimized using quantum-inspired evolutionary processes guided by clinical entropy and fitness-adaptive rotation.
- The mask is applied to select the subset of token embeddings (including the CLS token) for local fine-tuning, significantly reducing computational overhead.
- The client transmits the updated model weights and its optimized pruning mask back to the central server.
- The server aggregates model updates and pruning masks across clients to refine the global ViT and adaptive pruning strategy for the next round.

This iterative, privacy-preserving cycle enables efficient federated learning with adaptive token sparsification tailored to heterogeneous clinical data distributions. The main computation of the proposed framework is discussed in the following subsections.

#### 3.2.1. Client-Side Image Preprocessing

In order to ensure compatibility between the dataset and ViT-16/B model while enhancing discriminability, all images were subjected to a comprehensive preprocessing methodology consisting of three core steps, i.e., resolution normalization, augmentation, and intensity standardization.

Resolution Normalization:As the HyperKVASIR dataset comprises images with varying resolutions, ranging from 720×576 to 1920×1072 pixels due to differences in endoscopic equipment and acquisition protocols, all images are resized to a fixed resolution of 224×224 using bilinear interpolation to ensure compatibility with the ViT-16/B architecture. Moreover, the aspect ratio is maintained using zero padding to prevent anatomical distortion.

Augmentation: To prevent model overfitting and increase robustness, several augmentation steps are performed. These include spatial and affine transformations (rotation, flipping, etc.) to model variations in endoscopic viewpoint. To better reflect real clinical conditions, the images are augmented using brightness (±20%), contrast (±15%), and saturation (±10%) adjustments to account for varying illumination, together with Gaussian blur (σ≤1.0) and mild elastic deformations that mimic motion artifacts and tissue elasticity commonly observed during live endoscopic examinations.

Intensity Normalization:The pixel values of augmented RGB images are then scaled to the [0, 1] range and normalized with mean = [0.485, 0.456, 0.406] and standard deviation = [0.229, 0.224, 0.225]. These standard parameters are selected to allow stable convergence of ViT models [4].

#### 3.2.2. ViT Tokenization and Processing

Each client adopts the standard vision transformer, ViT-16/B, as a backbone model for feature extraction and learning. The three processing stages of the ViT are tokenization and patch embedding, transformer encoding, and final classification. These are discussed briefly as follows.

1. Image Tokenization and Patch Embedding: The preprocessed endoscopic image $I\in R^{224\times 224\times 3}$ is split into 196 patches, each having a size 16×16×3. Each patch $p_{i}$ is then flattened into a 1-D vector $X_{i}\in R^{768}$, followed by a linear projection onto d-dimensional embedding space as given by;
  (1) $$z_{i}=Ex_{i}+b_{E},\;z_{i}\in R^{d},$$
  where $E\in R^{d\times 768}$ is the projection matrix and $b_{E}$ is the local bias. In the ViT-16/B architecture, the embedding dimension is fixed at d=768 and maintained across all transformer encoder layers.
2. Positional Embedding: In order to preserve the spatial information of patch embedding, learnable position embeddings are added. A learnable CLS token $z_{\left[CLS\right]}\in R^{d}$ is prepended. The resulting input sequence $X_{0}$ for the transformer encoder is:
  (2) $$X_{0}=\left[z_{\left[CLS\right]};z_{1}+e_{1};\ldots ;z_{N_{p}}+e_{N_{p}}\right]$$
  where $X_{0}\in R^{(N_{p}+1)\times d}$
3. Transformer Encoder and Classification:
  The embedded token sequence $S_{0}$ is processed by a stack of L=12 transformer encoder layers. Each layer follows a pre-normalization design and consists of a multi-head self-attention (MHSA) block and a feed-forward network (FFN), with residual connections applied after each block. The forward computation at layer *ℓ* is given by:
  (3) $${\hat{S}}_{\ell }=S_{\ell -1}+\mathrm{MHSA}\left(\mathrm{LN}(S_{\ell -1})\right),$$
  (4) $$S_{\ell }={\hat{S}}_{\ell }+\mathrm{FFN}\left(\mathrm{LN}({\hat{S}}_{\ell })\right),$$
  where LN(·) denotes layer normalization.
  Multi-Head Self-Attention:
  Given the normalized input $\bar{S}\in R^{N\times d}$, the query, key, and value matrices are computed as:
  (5) $$Q=\bar{S}U_{Q},\;K=\bar{S}U_{K},\;V=\bar{S}U_{V},$$
  where $U_{Q},U_{K},U_{V}\in R^{d\times d}$ are learnable projection matrices. The attention mechanism employs H=12 parallel heads, each operating in a subspace of dimension $d_{h}=d/H$. For the *h*-th head, attention is computed as:
  (6) $${\mathrm{Attn}}_{h}=\mathrm{softmax}\left(\frac{Q_{h}K_{h}^{⊤}}{\sqrt{d_{h}}}\right)V_{h}.$$
  The outputs of all heads are concatenated and linearly projected to form the MHSA output:
  (7) $$\mathrm{MHSA}\left(Q,K,V\right)=\mathrm{Concat}\left({\mathrm{Attn}}_{1},\ldots ,{\mathrm{Attn}}_{H}\right)U_{O},$$
  where $U_{O}\in R^{d\times d}$.
  Feed-Forward Network (FFN):
  The FFN consists of two linear transformations with a GELU activation function:
  (8) $$\mathrm{FFN}\left(x\right)=W_{2}\;\mathrm{GELU}\left(W_{1}x+b_{1}\right)+b_{2},$$
  where $W_{1}\in R^{d\times 4d}$ and $W_{2}\in R^{4d\times d}$.
  Classification:
  After the final encoder layer, the representation of the class token $s_{\mathrm{cls}}^{\left(L\right)}\in R^{d}$ is used for classification:
  (9) $$\hat{y}=\mathrm{softmax}\left(g_{\psi }\left(s_{\mathrm{cls}}^{\left(L\right)}\right)\right),$$
  where $g_{\psi }$ denotes the learnable classification head. The model is trained using the categorical cross-entropy loss:
  (10) $$L=-\sum_{c}y_{c}\log\left({\hat{y}}_{c}\right).$$

### 3.3. Clinical Entropy-Guided Quantum Evolutionary Algorithm for Adaptive Token Pruning

This work proposes the Clinical-Entropy Guided Quantum Evolutionary Algorithm (CEQEA) as a novel optimization strategy for client-adaptive token pruning for ViTs in federated GI lesion classification scenarios. The CEQEA is an extension of the classical quantum evolutionary algorithm that incorporates three novel strategies, i.e., clinical-entropy–driven population scaling, fitness-aware individual rotation and token-level pruning constraints tailored to medical image analysis.

Let *N* denote the total number of ViT tokens (N=197 for ViT-16/B, including one [CLS] token). For each federated client *k*, CEQEA optimizes a binary pruning mask:

$$M\in {\left\{0,1\right\}}^{N},$$

where M=1 indicates retention of a token.

#### 3.3.1. Quantum Representation

In CEQEA, each decision variable is encoded as a qubit. The *j*-th qubit of the *i*-th individual at generation *t* is represented as:

$$|\psi_{i,j}\left(t\right)\rangle =\alpha_{i,j}\left(t\right)\;|1\rangle +\beta_{i,j}\left(t\right)\;|0\rangle ,$$

subject to the normalization constraint.

$$|\alpha_{i,j}{\left(t\right)|}^{2}+{\left|\beta_{i,j}\left(t\right)\right|}^{2}=1.$$

An individual quantum chromosome is defined as

(11) $$Q_{i}^{\left(t\right)}=\left[\begin{array}{cccc} \alpha_{i,1}^{\left(t\right)} & \alpha_{i,2}^{\left(t\right)} & \ldots & \alpha_{i,N}^{\left(t\right)}\\ \beta_{i,1}^{\left(t\right)} & \beta_{i,2}^{\left(t\right)} & \ldots & \beta_{i,N}^{\left(t\right)} \end{array}\right],\;i=1,\ldots ,P_{k},$$

where $P_{k}$ is the client-specific population size.

#### 3.3.2. Clinical Entropy–Guided Population Initialization

As shown in the dataset description, GI endoscopic data exhibit substantial inter-client variability arising from differences in disease prevalence, procedural focus, and case complexity across clinical centers. In particular, the class distribution at each client is often highly imbalanced, with a small number of dominant findings (e.g., normal mucosa or adequately prepared bowel) coexisting with visually subtle but clinically critical conditions such as early neoplasia or severe inflammatory disease. Such heterogeneity directly affects the difficulty of feature selection and token optimization within the proposed ViT framework.

To address variability caused by heterogeneous datasets and imaging protocols across federated clients, this work introduces a clinically motivated mechanism that adapts the evolutionary search capacity based on the diversity of each client’s local gastrointestinal (GI) dataset. Specifically, the evolutionary population size assigned to each client is dynamically adjusted according to the class distribution of its local dataset.

Let $D_{k}$ denote the local dataset of client *k*, which contains *C* diagnostic classes with a class distribution $P_{k}=\left\{p_{1},p_{2},\ldots ,p_{C}\right\}$. The clinical diversity of the dataset is quantified using Shannon entropy, defined as:

(12) $$H_{k}=-\sum_{j=1}^{C}p_{j}\log_{2}p_{j},$$

where $p_{j}$ represents the relative frequency of class *j* in $D_{k}$, computed as the number of samples belonging to class *j* divided by the total number of samples in the dataset. Since this entropy formulation relies on relative class proportions rather than absolute dataset size, it remains mathematically valid even when client datasets differ in size.

Based on this measure, the clinical-entropy–guided population size $P_{k}$ for the proposed CEQEA is defined as:

(13) $$P_{k}=\left\lfloor P_{B}\cdot \left(1+\alpha \cdot \frac{H_{k}}{\log_{2}C}\right)\right\rfloor ,$$

where $P_{B}$ denotes a base population size shared across all federated clients, and α∈{0,1} is a scaling factor that controls the allowable population expansion. Because the normalized entropy term $\frac{H_{k}}{\log_{2}C}$ lies within the range [0,1], the resulting population size $P_{k}$ is bounded within the interval $\left[P_{B},\left(1+\alpha \right)P_{B}\right]$.

This entropy-guided formulation enables each client to allocate evolutionary search capacity to its local clinical data distribution’s complexity while maintaining the federated learning paradigm by avoiding inter-client data sharing. Furthermore, since the entropy measure is based on normalized class proportions, it remains stable across datasets of varying sizes, although extremely small datasets may require smoothing or minimum-sample constraints to ensure reliable estimation.

After computing the entropy-adaptive population size $P_{k}$, the initial quantum population for iteration t=0 is constructed as:

(14) $$Q={\left[\begin{array}{c} Q_{1}^{\left(0\right)}\cdots Q_{P_{k}}^{\left(0\right)} \end{array}\right]}^{T}$$

where $Q_{i}$ is a quantum individual solution consisting of *N* qubits as represented in Equation (11). For ViT-16/B, the value N=197 corresponds to the total number of tokens in the ViT input sequence, including the CLS token.

#### 3.3.3. Quantum Observation

In the next step, the individual qubits of Q are probabilistically observed to generate a binary population. For qubit $q_{i,j}\in Q$, the observation $x_{ij}$ is obtained as:

(15) $$\begin{array}{c} x_{i,j}=\left\{\begin{array}{cc} 0 & \mathrm{if}\;r_{j}<{\left|\alpha_{i,j}\right|}^{2}\\ 1 & \mathrm{otherwise} \end{array}\right. \end{array}$$

where $\left(i\in 1,\cdots P_{k},j\in 1,\cdots ,N\right)$ and $r_{j}∼U\left(0,1\right)$ is a uniformly generated random number. The population matrix P at iteration *t* is represented as:

(16) $$\begin{array}{c} P^{\left(t\right)}=\left[\begin{array}{c} S_{1}^{\left(t\right)}\\ \vdots \\ S_{P_{k}}^{\left(t\right)} \end{array}\right]=\left[\begin{array}{ccc} x_{1,1} & \ldots & x_{1,N}\\ \vdots & \ddots & \vdots \\ x_{P_{k},1} & \ldots & x_{P_{k},N} \end{array}\right] \end{array}$$

where each vector $S_{i}^{\left(t\right)}\in {\left\{0,1\right\}}^{N}$ represents a candidate mask for token selection.

#### 3.3.4. Objective Function for Candidates Fitness Evaluation

This work adopts a composite fitness to evaluate the quality of each candidate pruning mask $m_{i}^{t}=S_{i}^{\left(t\right)}$ for client *k*. The fitness is computed as:

(17) $$f\left(S_{i}^{\left(t\right)}\right)=w_{1}\;A_{k}\left(S_{i}^{\left(t\right)}\right)+w_{2}\left(1-\frac{∥S_{i}^{\left(t\right)}{∥}_{0}}{N}\right)$$

The aforementioned fitness function jointly optimizes two competing objectives important in federated medical imaging analysis. The first term represents the diagnostic accuracy, in which $A_{k}\left(S_{i}^{\left(t\right)}\right)\in \left[0,1\right]$ denotes the classification accuracy on client *k*’s validation set using the pruned token set. $w_{1}\in \left[0,1\right]$ is the weight assigned to prioritize clinical accuracy. The second term represents the computational efficiency in terms of sparcity ratio $\frac{∥S_{i}^{\left(t\right)}{∥}_{0}}{N}$, where $∥S_{i}^{\left(t\right)}{∥}_{0}$ is the L0 norm, i.e., total number of non-zero elements (tokens retained) in the candidate mask and N=197 is the total tokens in ViT-16/B. This term is used to reward aggressive pruning. The weight $w_{2}=1-\beta_{1}$ is assigned a moderate value to balance accuracy and efficiency. In this work, the parameter $w_{1}=0.7$ was empirically selected via grid search on the holdout validation set.

After evaluating the fitness of each individual, the top $N_{b}$ chromosomes are selected as parents for the next generation. These parents then participate in uniform crossover, where each qubit of an offspring is selected from either parent with a fixed probability *p*.

#### 3.3.5. Federated Fitness Adaptive Quantum Rotation

The proposed CEQEA introduces a novel federated, fitness-adaptive quantum rotation mechanism that leverages both individual fitness performance and client-specific data characteristics. Let $b^{*}\left(t\right)$ denote the best solution at generation *t* with fitness $f^{*}=f\left(b^{*}\right)$. For individual *i* and qubit *j*, the rotation angle is computed as:

(18) $$\Delta \theta_{ij}\left(t\right)=\delta \cdot \mathrm{sign}\;\left(s_{ij}\left(t\right),b_{j}^{*}\left(t\right),f\left(fs_{i}\left(t\right)\right),f\left(b^{*}\left(t\right)\right)\right),$$

where δ is the rotation step, and the rotation direction is based on a binary logic.

(19) $$\mathrm{sign}\left(s_{ij},b_{j}^{*},f_{i},f^{*}\right)=\left\{\begin{array}{cc} +1, & \left(s_{ij},b_{j}^{*}\right)=\left(0,1\right),\;f_{i}<f^{*},\\ -1, & \left(s_{ij},b_{j}^{*}\right)=\left(0,1\right),\;f_{i}\geq f^{*},\\ +1, & \left(s_{ij},b_{j}^{*}\right)=\left(1,0\right),\;f_{i}\geq f^{*},\\ -1, & \left(s_{ij},b_{j}^{*}\right)=\left(1,0\right),\;f_{i}<f^{*},\\ 0, & \mathrm{otherwise}. \end{array}\right.$$

In the classical quantum-inspired evolutionary process, a fixed rotation step δ is applied to all individuals across all generations. This assumption is not effective in federated learning due to high variability in class distributions, procedural protocols, and disease prevalence. Therefore, as a key contribution, an entropy-aware rotation step size is proposed for each client and computed as follows:

(20) $$\begin{array}{c} \delta_{k}=\delta_{\mathrm{base}}\cdot \left(1+\gamma \cdot \frac{H_{\max}-H_{k}}{H_{\max}}\right) \end{array}$$

(21) $$\begin{array}{c} =\delta_{\mathrm{base}}\cdot \left(1+\gamma \cdot \left(1-\frac{H_{k}}{H_{\max}}\right)\right) \end{array}$$

where $\delta_{b}=0.05$ radians is the base rotation step, γ∈[0,0.5] is the entropy scaling factor, and $H_{\max}=\log_{2}C$ is the client’s maximum entropy. This entropy-guided rotation mechanism has clear clinical relevance. Specialized endoscopy centers, such as screening-focused colonoscopy units or disease-specific clinics, typically exhibit limited pathological variability, resulting in low-entropy datasets dominated by a small number of classes. Conversely, tertiary referral and academic centers manage a broad spectrum of conditions—including inflammatory lesions, dysplasia, malignancy, and post-interventional cases—leading to higher entropy due to increased class diversity and more balanced distributions. Accordingly, low-entropy clients employ larger rotation steps to accelerate convergence in relatively homogeneous data, whereas high-entropy clients adopt smaller rotation steps, enabling more conservative updates that preserve solution diversity and mitigate premature convergence in diagnostically complex settings.

The qubit update is then performed via a quantum phase flip gate:

(22) $$\left(\begin{array}{c} \alpha_{ij}^{\prime }\left(t\right)\\ \beta_{ij}^{\prime }\left(t\right) \end{array}\right)=\left(\begin{array}{cc} \cos(\Delta \theta_{ij}\left(t\right)) & -\sin(\Delta \theta_{ij}\left(t\right))\\ \sin(\Delta \theta_{ij}\left(t\right)) & \cos(\Delta \theta_{ij}\left(t\right)) \end{array}\right)\left(\begin{array}{c} \alpha_{ij}\left(t\right)\\ \beta_{ij}\left(t\right) \end{array}\right).$$

Each updated individual is expressed as:

(23) $$Q_{i}^{\prime }\left(t\right)=\left[\begin{array}{cccc} \left(\alpha_{i1}^{\prime },\beta_{i1}^{\prime }\right) & \left(\alpha_{i2}^{\prime },\beta_{i2}^{\prime }\right) & \cdots & \left(\alpha_{iN}^{\prime },\beta_{iN}^{\prime }\right) \end{array}\right].$$

Unlike traditional mutation operators in classical Genetic Algorithms, which randomly flip bits in a chromosome with a predefined probability, the quantum phase-flip gate operates on probability amplitudes within a quantum-inspired representation. This allows candidate solutions to maintain a probabilistic superposition of states rather than collapsing immediately into deterministic binary values. As a result, the phase-flip operation enables smoother exploration of the search space by adjusting probability amplitudes instead of performing abrupt bit mutations. This mechanism helps preserve population diversity and improves convergence stability, particularly in high-dimensional optimization problems such as token selection in ViT architectures.

#### 3.3.6. Federated Entropy Scaled Quantum Mutation

In the proposed CEQEA, the mutation rate is also controlled based on the clinical entropy of the participating client’s dataset. The clients with more diverse datasets perform minor mutation, whereas the clients with specialized datasets have a high mutation rate to perform more exploration. For qubit *j* in individual *i*, the mutation probability is computed as:

(24) $$P_{\mathrm{mut}}^{\left(ij\right)}=\eta \cdot \left(1-\frac{F_{i}}{F_{\max}}\right)\cdot \left(1-\frac{H_{k}}{H_{\max}}\right)\cdot S_{j}$$

where η∈[0,0.3] is the base mutation rate, $F_{i}$ is the fitness of individual *i*, $F_{\max}$ is the maximum fitness in the current generation, $H_{max}$ and $H_{k}$ are the maximum and clinical entropies of client *k* and $S_{j}\in \left[0,1\right]$ is the token stability score, computed from federated token selection frequencies as:

(25) $$S_{j}=\frac{1}{K}\sum_{k=1}^{K}\frac{N_{kj}^{s}}{N_{k}^{\mathrm{hf}}}$$

where $N_{kj}^{s}$ denotes the number of high-fitness masks selecting token *j* in the current population of client *k*, $N_{k}^{\mathrm{hf}}$ is the total number of high-fitness masks in the client *k* and *K* is the total number of federated clients.

The mutation is probabilistically triggered, performing the following quantum phase shift operation:

(26) $$q_{ij}^{\prime }=Z\cdot q_{ij}=\left(\begin{array}{cc} 1 & 0\\ 0 & -1 \end{array}\right)\left(\begin{array}{c} \alpha_{ij}\\ \beta_{ij} \end{array}\right)=\left(\begin{array}{c} \alpha_{ij}\\ -\beta_{ij} \end{array}\right)$$

where *Z* denotes the quantum phase-flip gate, which preserves the probability amplitude $|\alpha_{ij}{|}^{2}$ while introducing a phase shift to $\beta_{ij}$. The above steps of measurement, evaluation, and rotation updates are repeated for all individuals over several generations until stopping criteria (max generations or convergence) are met.

### 3.4. Integration of Proposed CEQEA in ViT-Assisted FL Framework for GI Endoscopy

The proposed CEQEA-based FL GI endoscopic framework consists of *K* healthcare institutions clients and a central server, which communicate through iterative federated learning rounds. Each round *t* comprises three core phases, with client-side entropy-guided token pruning via CEQEA serving as the critical step for achieving both computational efficiency and diagnostic accuracy in gastrointestinal image classification. These phases are detailed as follows.

#### 3.4.1. Phase 1: Server Initialization and Broadcast

During this phase, the central server maintains the parameters of the global ViT-16/B model $\theta^{t}$ and a global token selection mask $m^{t}$. A subset $S_{t}\subseteq \left\{1,2,\ldots ,K\right\}$ of clients is selected, and the tuple $\left(\theta^{t},m^{t}\right)$ is broadcast to each selected client.

#### 3.4.2. Phase 2: Local Client Update with CEQEA-Based Token Pruning

Each client $k\in S_{t}$ performs the following steps on its private gastrointestinal endoscopic dataset $D_{k}$.

**Figure 4:**
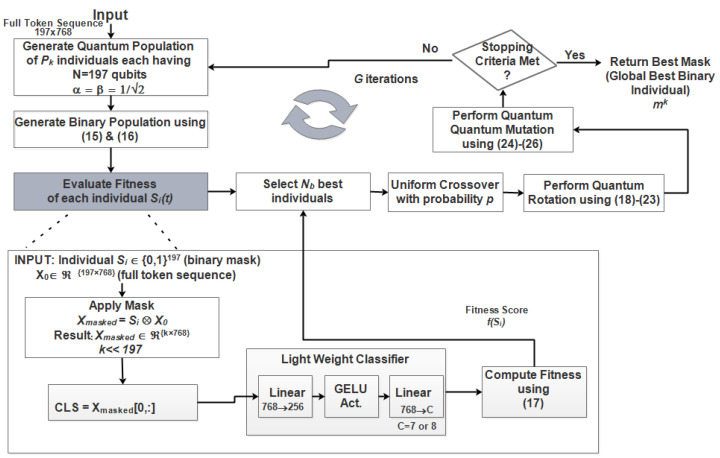
Main Steps of the proposed CEQEA for token pruning.

Token Preparation and CEQEA-Based Pruning: The step-by-step process of converting an input endoscopic image into a token sequence and applying the CEQEA for adaptive token pruning is outlined below:

1. Input Image: A preprocessed endoscopic image $I\in D_{k}$ of size 224×224×3 is received.
2. Patch Formation: The image is divided into non-overlapping patches of size 16×16×3, yielding $\left(\frac{224}{16}\right)\times \left(\frac{224}{16}\right)=196$ patches.
3. Patch Flattening: Each patch is flattened into a 1-D vector of length 16×16×3=768.
4. Embedding: Flattened patches are projected into 768-dimensional learnable embeddings via a linear projection layer.
5. Patch Embedding Output: The output is a tensor of shape 196×768.
6. [CLS] Token Addition: A learnable classification token of dimension 768 is prepended, resulting in a sequence length of 197 (1 [CLS] token + 196 patch tokens).
7. Positional Embeddings: Sinusoidal positional embeddings are added to provide spatial context, producing the final input sequence $X_{0}\in R^{197\times 768}$ for the transformer encoder.
8. CEQEA Token Selection: This core step generates a client-specific binary pruning mask $m_{k}\in {\left\{0,1\right\}}^{197}$ using CEQEA. The [CLS] token is always retained ($m_{k}\left[0\right]=1$). Figure 4 shows the flowchart of computation steps of CEQEA algorithm which proceeds as follows:
  - Clinical Entropy Calculation: Compute local clinical entropy $H_{k}$ from class distribution of $D_{k}$. It is important to note that the clinical entropy measure in the proposed framework is computed from the class distribution of each client’s dataset rather than from individual image patches. Therefore, common endoscopic artifacts such as motion blur, bubbles, or specular reflections do not directly affect the entropy calculation. Instead, entropy only regulates the evolutionary search capacity of the CEQEA optimizer. The actual token selection is guided by the Vision Transformer’s attention responses and classification loss, which typically assign lower importance to noisy or non-informative regions. As a result, artifact-heavy patches are generally identified as low-utility tokens and pruned during optimization.
  - Quantum Population Initialization: Compute population size $P_{k}$ using Equation (13). Generate a quantum population $Q_{i}^{t}$ of $P_{k}$ individuals, each with N=197 qubits initialized per Equation (11).
  - Classical Observation: Generate binary population P via quantum state observation (Equation (15)).
  - Fitness Evaluation: For each binary individual $s_{i}\in P$:
    (a) Apply $s_{i}$ as a mask to select tokens from $X_{0}$.
    (b) Extract the masked [CLS] token embedding $Z_{\mathrm{CLS}}\in R^{768}$.
    (c) Train a lightweight MLP classifier having two linear layers (68→256→C) with GELU activation and dropout between for 1–3 epochs on the client’s training split using the masked CLS features.
    (d) Compute fitness $F(s_{i})$ using Equation (17).
  - Evolutionary Operations: Over *G* generations:
    – Perform fitness-adaptive quantum rotation using client-adaptive step size $\delta_{k}$.
    – Apply entropy-controlled quantum mutation with probability $P_{\mathrm{mut}}^{\left(ij\right)}$.
    – Retain top $N_{b}$ elites; perform uniform crossover with probability $p_{c}$.
  - Mask Output: After *G* generations, output the best binary individual as the optimized pruning mask $m_{k}$.
9. Transformer Encoder Processing: Apply the mask via element-wise multiplication to the token sequence:
  $${\tilde{X}}_{0}=m_{k}⊙X_{0}$$
  The pruned sequence is processed through L=12 transformer encoder layers, significantly reducing the computational complexity of the Multi-Head Self-Attention (MHSA) step.
10. Final Classification: The transformed [CLS] token output (768-dimensional) is passed through the classification head $h_{\phi }$ to produce diagnostic logits for gastrointestinal disease classes.

Local Fine-tuning: The client fine-tunes the ViT classification head and optionally the last transformer layers using the masked token features for *E* local epochs, minimizing the cross-entropy loss over $D_{k}$.
Upload to Server: Each client $k\in S_{t}$ transmits its updated local model parameters $\theta_{k}^{t+1}$ along with its optimized pruning mask $m_{k}$ and clinical entropy $H_{k}$ back to the central server.

#### 3.4.3. Phase 3: Server Aggregation

The server performs a two-fold aggregation:

- Model Parameter Aggregation: Using federated averaging:
  (27) $$\theta^{t+1}=\sum_{k=1}^{K}\frac{n_{k}}{n}\theta_{k}^{\left(t+1\right)}$$
  where $n_{k}=\left|D_{k}\right|$ and $n=\sum_{k}n_{k}$.
- Token Mask Aggregation: Using clinical entropy-weighted fusion:
  (28) $$s_{j}=\sum_{k=1}^{K}\left(\frac{H_{k}}{\sum_{l=1}^{K}H_{l}}\right)\cdot m_{j}^{\left(k\right)},\;m_{j}^{\mathrm{global}}=\left\{\begin{array}{cc} 1 & \mathrm{if}\;s_{j}\geq \eta \\ 0 & \mathrm{otherwise} \end{array}\right.$$
  with consensus threshold η=0.6. This ensures a token is retained globally only if it is selected by a weighted majority of clients, prioritizing consensus from institutions with higher clinical diversity.

The server then broadcasts the updated global model $\theta^{t+1}$ and aggregated global mask $m^{t+1}=m^{\mathrm{global}}$ to all clients for the next federated round. The pruning mask $m_{k}$ is primarily introduced to reduce the computational cost of federated training by eliminating redundant image tokens during local client optimization. By limiting the number of processed tokens, the proposed CEQEA framework decreases both computational workload and communication overhead in decentralized training environments. Although the mask is optimized during the training phase, the resulting token-selection pattern can also be applied during inference, enabling faster prediction while preserving diagnostically relevant visual information. This property may be beneficial for real-time clinical deployment where computational resources are limited. Prior to processing, each client performs dataset-specific preprocessing—including resizing, normalization, and augmentation tailored to gastrointestinal endoscopic imagery—on local images.

## 4. Results and Discussion

### 4.1. Experimental Setup

The proposed federated learning framework, illustrated in Figure 3, comprises a central aggregation server and K=3 clinical client nodes simulating distinct healthcare institutions (e.g., oncology centers, screening clinics, tertiary hospitals). The participation of the clients was randomized; such that only (any) two of the three clients participated in the federation process. Each client independently executes the CEQEA to perform adaptive token pruning and locally fine-tunes the ViT-16/B on its private, secure GI endoscopic dataset, ensuring that all raw patient data remain on-premises to preserve privacy and comply with medical data regulations. Upon completing local optimization in each communication round, clients upload only their encrypted model updates and optimized pruning masks to a secure cloud storage. The central server retrieves these updates, performs clinical entropy-weighted aggregation of both model parameters and pruning masks, and distributes the refined global model along with the consensus pruning mask back to all clients for subsequent rounds.

All experiments were conducted within a secure, isolated network environment emulating a real-world multi-institution research collaboration. Each client node was equipped with an Intel Core i7-12700K CPU (12 cores, 3.6 GHz), an NVIDIA RTX 3080 GPU (10 GB VRAM), 32 GB DDR4 RAM, and a 1 TB NVMe SSD, running Ubuntu 22.04 LTS. The central aggregation server maintained comparable hardware with enhanced networking and storage capabilities to ensure efficient orchestration. Federated learning coordination—including round scheduling, client sampling, secure aggregation, and model synchronization—was managed using the Flower framework, extended with custom modules for CEQEA-based token pruning and entropy-aware aggregation.

### 4.2. Training Configuration

To mimic real-world clinical data heterogeneity, the upper and lower gastrointestinal tract portions of the HyperKVASIR dataset were partitioned across K=3 federated clients using a Dirichlet distribution-based allocation (Dir(α)) [23], a widely adopted approach for simulating label distribution skew in federated learning benchmarks [24]. The concentration parameter α controls the degree of label skewness, with lower values (α<1.0) producing stronger heterogeneity across clients. Each client was assigned a distinct clinical profile (e.g., upper GI–focused, lower GI–focused, or mixed) through non-IID partitioning, ensuring that no single client possessed a uniformly distributed subset of all diagnostic classes. Client participation was randomized in each round with a probability of $\frac{2}{3}$ to emulate realistic institutional availability constraints while preserving computational efficiency and encouraging diversity in federated updates.

It is important to note that the HyperKVASIR dataset provides annotated endoscopic images without patient-level identifiers. Consequently, dataset partitioning and splitting were performed at the image levelrather than the patient or procedure level. To reduce the potential risk of data leakage, the curated subset used in this study consists of distinct annotated images representing different visual observations captured during endoscopic procedures rather than sequential frames extracted from videos. Furthermore, the dataset contains images from multiple clinical examinations, with substantial variability in tissue appearance, illumination conditions, and viewing angles, reducing the likelihood of nearly identical images appearing across the training and evaluation subsets.

The Federated Averaging (FedAvg) algorithm was used to aggregate local model updates, requiring at least two client updates per round. A Dirichlet allocation with α=0.5 was applied to partition samples of each diagnostic class into shards distributed among clients, simulating realistic imbalances in gastrointestinal lesion distributions across clinical sites. Within each client’s dataset, an 80:20 hold-out split was applied to create training and validation subsets used for local model optimization.

Transfer learning was employed to initialize the Vision Transformer backbone (ViT-16/B) using ImageNet-pretrained weights. Hyperparameters were selected through grid search on the client-side validation splits to balance computational efficiency and convergence stability, as summarized in Table 1. In accordance with federated learning best practices [25], a batch size of 8 and one local epoch per communication round were used to mitigate communication overhead while maintaining stable model updates. The learning rate was set to $1\times 10^{-5}$, consistent with prior federated ViT training benchmarks [26]. During each of the 50 global communication rounds, participating clients performed one local training epoch using stochastic gradient descent (SGD) with momentum. After local training, the updated model parameters and optimized CEQEA pruning masks were transmitted to the server for clinical-entropy–weighted aggregation, after which the updated global model was redistributed to clients for the subsequent round.

Several key metrics were tracked across rounds: training accuracy, training loss, confusion matrices, CEQEA fitness progression, token retention rates, and per-class diagnostic performance. These metrics facilitated analysis of both convergence behavior and the adaptive token pruning efficacy under heterogeneous data conditions.

### 4.3. Results of HyperKVASIR Lower GI Tract Dataset

**Figure 5:**
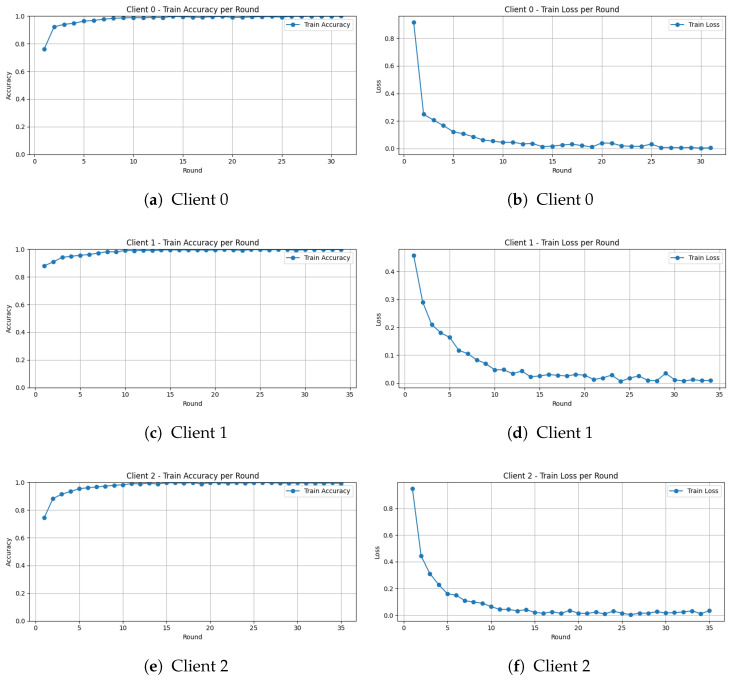
Local training accuracy and Loss plots for three clients across federated learning rounds for the HyperKVASIR Lower GI Tract dataset.

Figure 5 shows the training accuracy and loss of the proposed CEQEA framework for three clients as a function of federated rounds for the lower GI tract subset of the HyperKVASIR dataset. The plots clearly reveal a convergent and stable behavior of the proposed methodology for all the clients under realistic non-IID conditions. All clients exhibit a steep learning curve during federated training. For Client 0, the training accuracy increases rapidly from approximately 74% in the first communication round to nearly 99% by the 10th round, indicating fast convergence. Clients 1 and 2 demonstrate highly consistent learning patterns, achieving comparable accuracy levels at a similar stage of training. This synchronized convergence across clients suggests stable optimization despite heterogeneous local data distributions.

The training loss curves closely mirror the observed accuracy trends, showing a sharp, monotonic decrease across successive federated rounds. For Client 0, the training loss begins at approximately 0.94 in the first round and drops below 0.1 by the 10th round, thereafter asymptotically approaching zero in subsequent iterations. Clients 1 and 2 follow an almost identical trajectory, confirming uniform convergence behavior across participating institutions.

**Figure 6:**
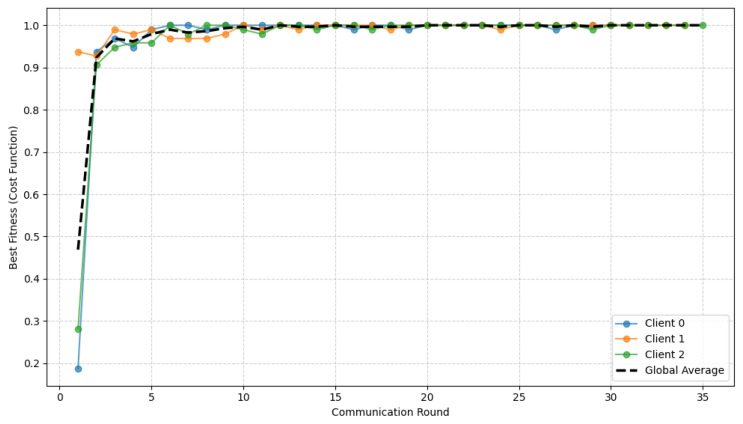
CEQEA Best Fitness (Client-Wise, Global Average) as a function of Communication Rounds for HyperKVASIR Lower GI Tract Dataset.

Figure 6 illustrates the evolution of the CEQEA best fitness as a function of the communication rounds. The fitness curves for all participating clients exhibit a rapid, consistent increase, attaining the optimal value of 1.0 within the first 10 communication rounds and maintaining stability in subsequent iterations. This early saturation indicates the fast convergence of the underlying evolutionary search process. Notably, the global average fitness curve closely aligns with the individual client trajectories, demonstrating strong synchronization across the federation despite heterogeneous local data. The resulting convergence is both rapid and robust, underscoring how entropy-aware evolutionary optimization can enhance federated learning efficiency and generalization.

**Figure 7:**
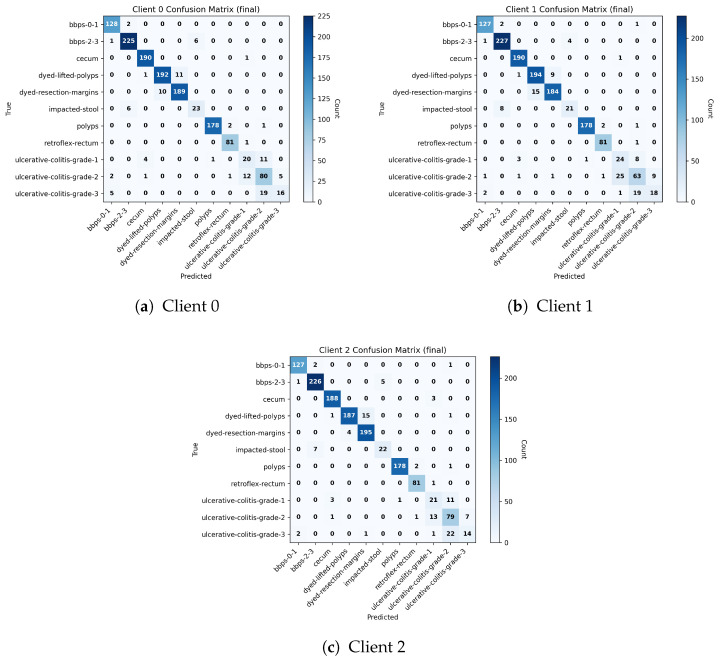
Test Confusion Matrices of three clients using the updated global model on the KVASIR Lower GI Tract dataset.

Figure 7 presents the final test confusion matrices obtained by the three federated clients on the Lower GI tract subset of the HyperKVASIR dataset. Across all clients, the matrices show a strong concentration of predictions along the main diagonal, indicating reliable and consistent class discrimination after federated training. Clear anatomical and procedural categories, such as cecum, polyps, retroflex rectum, and bowel preparation (bbps) classes, are recognized with high confidence. Most observed misclassifications occur between clinically related categories, particularly between different ulcerative colitis severity grades and between dyed-lifted polyps and dyed resection margins. These confusions are clinically understandable due to overlapping visual characteristics and subtle boundary differences. Importantly, errors rarely spill into unrelated classes, suggesting that the learned representations remain clinically coherent. This behavior reflects the effectiveness of the CEQEA-based entropy-aware pruning strategy, which selectively preserves informative tokens while suppressing redundant visual cues, allowing each client to adapt its feature representation to its local data characteristics. It is important to note that the CEQEA-based pruning mechanism does not enforce a fixed token selection for specific diagnostic classes. Instead, token importance is evaluated during training according to its contribution to classification performance. For visually similar categories such as dyed-lifted polyps and dyed resection margins, the optimization process may initially retain overlapping regions due to shared visual patterns. However, as training progresses, the evolutionary search identifies more discriminative token subsets that capture subtle structural cues, allowing the model to adaptively emphasize class-specific boundaries and morphological characteristics.

Table 2 summarizes the micro- and macro-averaged performance metrics derived from the confusion matrices of the three clients. These metrics have been computed as an average of 5 Monte Carlo simulations of the pipeline with different random seeds. The micro-averaged results show strong overall performance, with accuracy, precision, recall, and F1-score reaching 92.77%, alongside a very high specificity of 99.25%, indicating a low false-positive rate across all classes. The macro-averaged metrics, while slightly lower, remain stable and reflect balanced learning across both frequent and less-represented categories. This gap between micro- and macro-level performance highlights the inherent class imbalance and variability in visual complexity within GI endoscopic data. The entropy-aware CEQEA framework plays a key role in mitigating these challenges by adapting the pruning process to each client’s clinical diversity. Clients with more heterogeneous data retain a broader set of tokens to capture subtle patterns, whereas more homogeneous datasets converge toward compact, stable representations. As a result, the framework achieves strong generalization across institutions while maintaining sensitivity to clinically important but less prevalent conditions.

### 4.4. Results of HyperKVASIR Upper GI Tract Dataset

**Figure 8:**
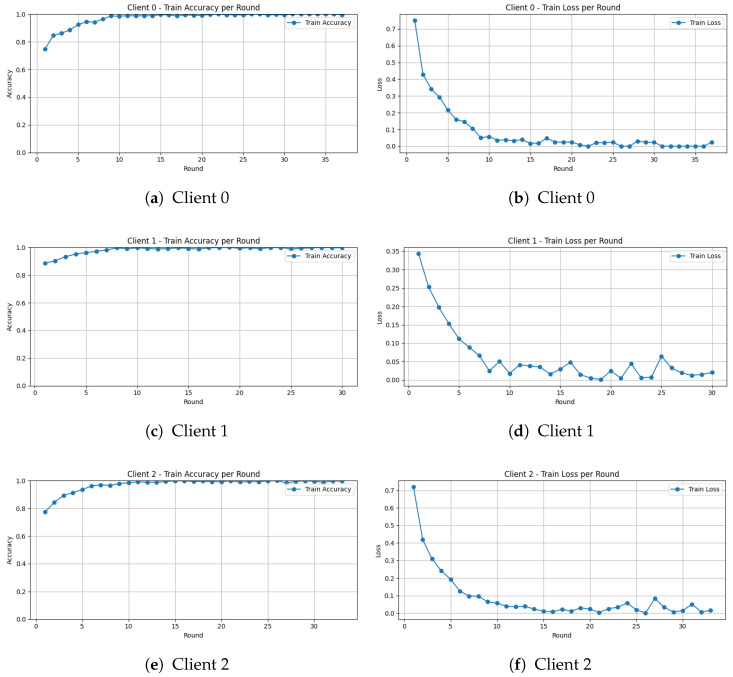
Local training accuracy and Loss plots for three clients across federated learning rounds for the HyperKVASIR Upper GI Tract dataset.

In Figure 8, the training accuracy and loss are illustrated for the three clients across federated learning rounds on the HyperKVASIR upper GI tract dataset. All clients exhibit a sharp and stable learning behavior, with training accuracy rapidly reaching approximately 99–100% within the first 10 rounds. This fast convergence indicates that the model effectively captures clinically meaningful visual cues early in training, including mucosal patterns and lesion-specific characteristics. The similar convergence trends across clients highlight the robustness of the learned representations and the minimal inter-client variability, despite the data being distributed across a federated setting. Consistently, the training loss shows a steep decline during the initial rounds, followed by stabilization near zero, further confirming stable optimization and comparable performance across all clients.

**Figure 9:**
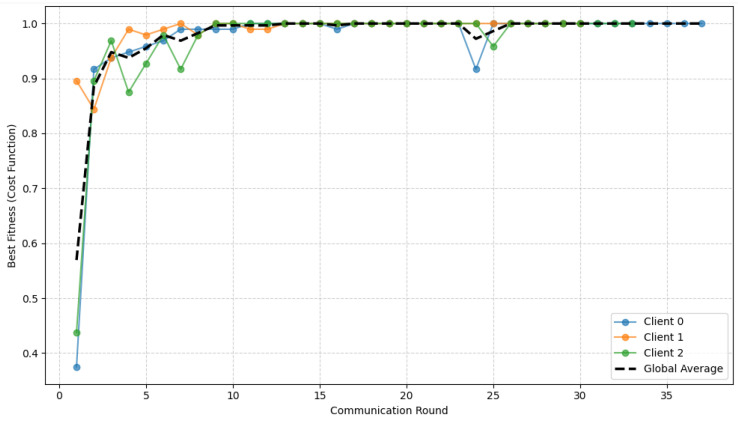
CEQEA Best Fitness (Client-Wise, Global Average) as a function of Communication Rounds for HyperKVASIR Upper GI Tract Dataset.

In a manner analogous to the lower GI tract dataset, the convergence behavior of the proposed CEQEA is analyzed in Figure 9. The best fitness curves for individual clients, along with their global average, show consistent, rapid improvement over federated learning rounds. The fitness value approaches the optimal level (1.0) within the initial rounds and remains stable thereafter, indicating fast convergence and robust optimization. This behavior confirms that the entropy-aware CEQEA effectively balances exploration and exploitation across heterogeneous clients, enabling reliable and synchronized convergence in the federated Upper-GI setting.

**Figure 10:**
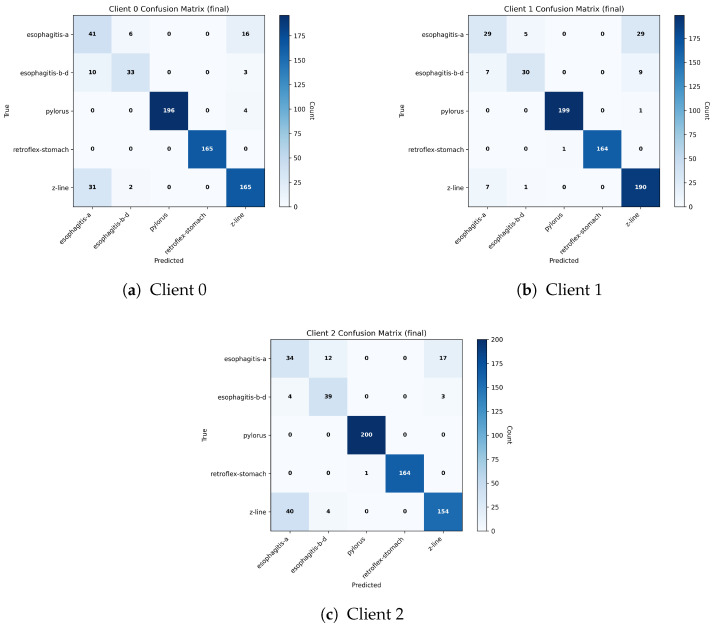
Test Confusion Matrices of three clients using updated global model on KVASIR Upper GI Tract dataset.

In Figure 10, the confusion matrices are shown for all three federated clients on the HyperKVASIR Upper GI tract test dataset. The proposed framework achieves strong diagonal peformance, indicating accurate class-wise predictions. Almost perfect predictions are obtained by the pylorus and retroflex-stomach classes. The z-line, esophagitis-a, esphagitis-b-d classes show minor misclassifications due to their visual similarities and gradual pathological transitions in endoscopic views. Overall, all classes exhibit diagonal dominance and consistent, reliable performance across clients, highlighting the robustness of the proposed federated framework under data heterogeneity.

In Table 3, the micro- and macro-averaged evaluation metrics are reported for all three clients. The proposed framework achieves about 90% mean microaveraged accuracy, precision, recall, and F1-score. These values show a diagnostically reliable and robust performance despite non-IID partition and visual similarities of some classes. A high mean micro-averaged specificity of 97.64 is achieved, which is very important to minimize false positives in clinical settings. The macro-averaged results are also competitive and indicate balanced performance across classes. These results demonstrate the effectiveness of the proposed CEQEA methodology, which can extract discriminative tokens in complex, high-entropy clinical settings while enabling faster convergence in more homogeneous datasets.

### 4.5. Discussion

The performance metrics and confusion matrices across both upper and lower GI datasets underscore the clinical coherence of the proposed CEQEA-based federated framework. The model’s robust performance in identifying procedural landmarks (e.g., pylorus, cecum) and diagnostic targets (e.g., polyps) indicates significant potential for real-time procedural guidance. From a clinical perspective, the high specificity observed across all federated clients—99.23% for the Lower GI and 97.64% for the Upper GI datasets—is a critical performance indicator. In endoscopic screening, minimizing false-positive rates is essential to prevent unnecessary biopsies or excessive follow-up intervals, thereby maintaining overall clinical efficiency and reducing patient burden.

Minor classification overlaps observed in the confusion matrices—specifically within the ulcerative colitis (UC) severity grading and post-resection margin assessment—are diagnostically relevant. These misclassifications occur primarily at the boundaries of disease severity, mirroring the inter-observer variability often documented among human endoscopists. By maintaining high diagnostic precision in these transitional zones, the framework provides an objective baseline that could, in future iterations, mitigate the subjective nature of manual inflammation scoring. Importantly, errors rarely extend to anatomically unrelated classes, suggesting that the learned feature representations remain clinically consistent.

The significance of the entropy-guided adaptive pruning strategy lies in its ability to reconcile heterogeneous data environments. Unlike centralized models that rely on static pruning policies, the CEQEA framework dynamically optimizes token retention based on the unique visual entropy of each clinical client. This allows the model to prioritize complex lesion features in specialized oncology centers while streamlining computational load in high-volume screening clinics. Consequently, the framework achieves the dual objective of maintaining high diagnostic sensitivity for clinically critical lesions while ensuring the operational leaness required for deployment on standard medical workstation hardware.

Quantitatively, the framework achieves a mean micro-averaged accuracy of 92.33% for the Lower GI dataset and 90.19% for the Upper GI dataset, indicating stable diagnostic performance across distributed clinical data sources. The adaptive token selection mechanism contributes to this stability by dynamically identifying informative image regions during training—specifically focusing on mucosal texture, vascular patterns, and lesion boundaries.

In summary, these results demonstrate that the CEQEA framework can effectively learn clinically relevant visual representations from diverse, non-IID endoscopic data. The framework facilitates privacy-preserving collaborative learning across institutions without compromising diagnostic reliability, providing a scalable solution for integrating advanced AI-driven decision support into clinical practice.

### 4.6. Performance Comparison with Existing Works on HyperKVASIR

Table 4 presents a comparative performance analysis of the proposed federated learning framework incorporating CEQEA-based token pruning within a ViT-16/B backbone. For a fair and meaningful comparison, only studies that report GI image classification results on the HyperKVASIR dataset are considered. It is important to note that reported results from prior work are presented for contextual reference rather than as strictly controlled benchmarks, as differences in dataset splits, preprocessing pipelines, and computational environments prevent direct equivalence. Most of the published works on GI classification using endoscopic images rely on centralized training paradigms using CNN-based or ViT-based architectures. In [6], the release of the HyperKVASIR dataset was carried out along with the evaluation of conventional CNNs such as ResNet and DenseNet in a fully centralized setting. A reasonable accuracy (≈91%) was achieved; however, the analysis relied on extensive data aggregation and lacked a mechanism to address inter-class imbalance. The subsequent works were focused on improving the feature discrimination of centralized learning using modified architectures and ensembles of CNN models. In [27], an EfficientNet-B0 and EfficientMix augmentation is proposed, achieving high accuracy (97.99%) and sensitivity (98.1%).

In [28], the authors proposed an ensemble of Inception, Xception, and ResNet101 models augmented with minimum Redundancy Maximum Relevance (mRMR) feature selection, reporting very high accuracy and specificity. Feature modeling strategies were also investigated in some works. Ref. [29] integrated CapsNet with VGG features to model spatial relationships, though performance remained comparatively modest, particularly in precision and recall. In [30], a lightweight MobileNetV2 architecture was proposed, prioritizing efficiency over accuracy, resulting in lower F1-scores. These studies highlight the trade-off between model compactness and diagnostic reliability in GI classification tasks. In recent work, ViT models have been investigated for GI image analysis. The authors in [31] proposed a custom ViT architecture with hybrid attention windows to capture multi-scale contextual features; however, limited accuracy was achieved, possibly due to insufficient token optimization. In [32], an ensemble of MobileViT variants was proposed to improve efficiency, but still exhibited reduced sensitivity and F1-scores. These studies indicate challenges in capturing subtle pathological differences across classes. Although significant research effort has been devoted to GI image classification using centralized CNN- and ViT-based architectures on the HyperKVASIR dataset, federated learning remains largely unexplored in this domain. To the best of our knowledge, no prior work has reported federated ViT results on the HyperKVASIR dataset, highlighting a clear gap that the proposed CEQEA-based framework addresses. A recent federated study leveraged subsets of HyperKVASIR for privacy-preserving representation learning within a foundation model framework, achieving a coarse-grained accuracy of approximately 81.3%. However, that work focused on generic feature learning rather than lesion-level or tract-specific classification and did not report clinically relevant performance metrics such as sensitivity, specificity, or F1-score. To the best of our knowledge, the proposed CEQEA-based framework is the first work to perform full multi-class GI classification on HyperKVASIR in a federated setting, reporting comprehensive clinical metrics, including precision, sensitivity, specificity, and F1-score. The results of Table 4 demonstrate that the proposed CEQEA-based FL framework achieves performance comparable to strong centralized baselines, while avoiding direct data sharing. By combining federated learning with clinically informed token selection, the method supports privacy-preserving collaboration across hospitals while maintaining reliable sensitivity to diverse gastrointestinal pathologies—an essential requirement for real-world deployment in modern endoscopy workflows.

**Table 4:** Contextual Comparison of proposed work with some representative Convolutional Neural Networks (CNN)-, Vision Transformer (ViT)-, and Federated Learning (FL)-based methods evaluated on the HyperKVASIR dataset. The proposed CEQEA framework is evaluated in a federated setting with three clients.

Work	Category	Architecture	Acc. (%)	Prec. (%)	Sen. (%)	Spec. (%)	F1 (%)
[6]	Centralized	ResNet/DenseNet	91.0	-	-	-	-
[27]	Centralized	EfficientNetB0 + EffMix	97.99	97.0	98.1	-	97.0
[29]	Centralized	VGG + CapsNet	85.0	54.0	55.0	-	55.0
[30]	Centralized	MobileNetV2	90.44	-	-	-	65.42
[28]	Centralized	Ensemble (Inception, Xception, ResNet101) + mRMR	98.4	98.0	99.0	99.0	98.0
[31]	Centralized	Custom ViT with Hybrid Windows	86.81	-	-	-	-
[32]	Centralized	Ensemble (MobileViT_XS + MobileViT_V2)	91.98	69.0	63.0	-	64.0
[33]	FL (Pretraining)	Federated Foundation Model	81.3	-	-	-	-
This work	FL	ViT-16/B + CEQEA Token Pruning	92.33	92.33	92.33	99.23	92.33

### 4.7. Ablation Study: Convergence and Complexity Analysis

This subsection details the convergence behavior as well as sheds light on the computational and communication complexities.

#### 4.7.1. Learning Efficiency: Convergence Behavior

**Figure 11:**
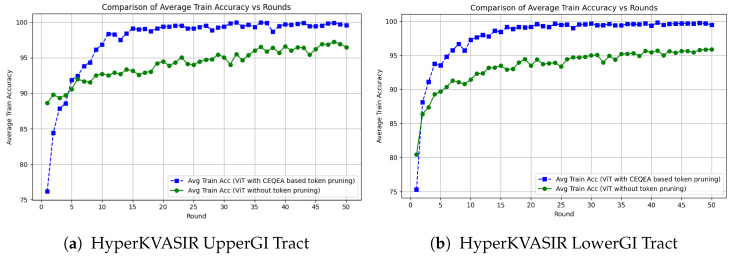
Average training accuracy of three clients using the Proposed CEQEA-ViT Token Pruning framework versus standard ViT-16/B based FL pipeline on HyperKVASIR Upper and Lower GI Tract datasets.

In addition to literature-based comparisons, an internal baseline experiment was conducted using a standard ViT-16/B model without token pruning within the same federated training pipeline. Both the baseline and the proposed CEQEA-based framework were trained and evaluated using identical dataset partitions, preprocessing procedures, training epochs, and evaluation metrics to ensure a fair and controlled comparison. Figure 11a,b corresponding to the Upper-GI and Lower-GI HyperKVASIR datasets illustrate the evolution of average training accuracy across federated communication rounds for both approaches.

Across both anatomical tracts, the CEQEA-enabled ViT consistently converges faster and more stably than the standard ViT-FL baseline. On the HyperKVASIR Upper-GI dataset, the CEQEA-pruned ViT achieves an average training accuracy of 98% across approximately 8–10 federated rounds, whereas the standard ViT-based federated pipeline requires 15–18 rounds to reach the same accuracy. This corresponds to a ≈40–50% reduction in communication rounds required for high-performance convergence. Moreover, the CEQEA-based model stabilizes near 99–100% accuracy shortly after convergence, while the baseline ViT exhibits significant oscilations before settling. A similar trend is observed on the HyperKVASIR Lower-GI dataset. The proposed CEQEA-based ViT surpasses 97% average accuracy by round 10, while the non-pruned ViT requires approximately 18–20 rounds to approach comparable performance. Throughout the training, the CEQEA-pruned ViT shows a consistent 3–4% accuracy gap v.s. the standard ViT FL pipeline. This fast and stable convergence behaviour is attributed to the proposed CEQEA, an entropy-aware and adaptive token selection mechanism that explicitly discards low-information tokens during early training, enabling the ViT to focus on clinically salient regions such as mucosal textures, anatomical landmarks, and lesion boundaries. This targeted token reduction acts as an input-adaptive regularization mechanism, leading to faster convergence and reduced computational overhead.

**Figure 12:**
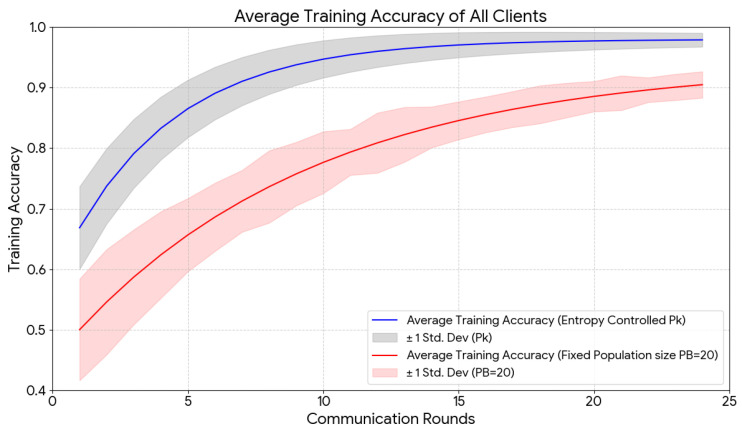
Average training accuracy comparison of Fixed Population Size $P_{B}=20$ and clients entropy controlled population size $P_{K}$ for HyperKVASIR Lower GI Tract.

Figure 12 illustrates the average training accuracy of the three clients with ±1 standard deviation on the Lower-HyperKVASIR dataset under two population configurations: a fixed population size ($P_{B}=20$) and the proposed entropy-guided population size ($P_{k}$). The results show that the entropy-guided population converges faster and exhibits lower client-to-client variability, resulting in more consistent and stable training performance.

#### 4.7.2. Computational Complexity

The primary source of ViT complexity in the federated setting is the Multi-Head Self-Attention (MHSA) mechanism, which exhibits quadratic computational complexity of $O(N^{2}\cdot d)$, where *N* is the token sequence length (N=197 for ViT-16/B) and *d* is the embedding dimension (d=768). In each local training round, a standard federated ViT must process all *N* tokens, incurring the full $O(N^{2}\cdot d)$ cost per attention operation. The proposed CEQEA framework prunes the token sequence adaptively for each client, retaining only K≪N clinically salient tokens. Consequently, the per-client MHSA complexity reduces to $O(K^{2}\cdot d)$. Experimental evaluation on the HyperKVASIR dataset shows that the average number of retained tokens after CEQEA pruning is K≈90, with a maximum of K≈120. For N=197 and K=120, this yields a theoretical reduction factor of approximately $\left(\frac{K^{2}}{N^{2}}\right)\approx 37\%$ in the dominant transformer encoder cost, corresponding to a 63% reduction in attention operations.

The computational overhead of CEQEA itself is O(P·G·C), where *P* is the entropy-adaptive population size, *G* is the number of evolutionary generations (set to 10 in this work), and *C* is the cost of fitness evaluation per individual. Importantly, this overhead is incurred only once per federated communication round and is largely offset by the substantial savings in token processing across multiple local training epochs. Empirical measurements indicate that the CEQEA runtime overhead remains below 0.05% of total training time.

#### 4.7.3. Communication Complexity

In a conventional FL-ViT pipeline, each client transmits the full ViT model parameters θ (≈86 million parameters for ViT-16/B) to the central server at each round. While the proposed framework does not compress model size, it achieves significant communication savings by accelerating convergence. As shown in the results, the CEQEA-pruned ViT achieves >98% accuracy in approximately 10 communication rounds on the HyperKVASIR dataset, whereas the unpruned federated ViT baseline requires 15–20 rounds to achieve comparable performance. This reduction in required communication rounds directly translates to a proportional decrease in total data transmission, which is often more impactful than per-update compression in bandwidth-constrained clinical networks.

#### 4.7.4. Overall Efficiency Gains

By combining adaptive token pruning with clinical-entropy-guided evolutionary search, the CEQEA framework achieves a balanced trade-off between computational efficiency and diagnostic accuracy. The reduction in token count lowers local GPU memory usage and accelerates forward/backward passes during client-side training. Furthermore, the faster convergence reduces the number of synchronization rounds, decreasing both communication latency and aggregate network overhead. These efficiency gains make the framework particularly suitable for real-world federated learning scenarios involving heterogeneous, non-IID medical data across resource-constrained healthcare institutions.

The complete quantitative analysis of complexity is summarized in Table 5.

The performance and complexity analyses demonstrate that the proposed CEQEA effectively reduces the computational and communication bottlenecks in ViT-based FL for GI classification. The framework performs client-adaptive token pruning, significantly lowering the quadratic complexity of multi-head self-attention while maintaining high diagnostic accuracy. CEQEA reduces attention complexity by approximately 79.1% and total communication overhead by up to 46.7% across the HyperKVASIR Upper and Lower GI tract datasets, demonstrating its suitability for federated medical imaging with heterogeneous, non-IID data distributions.

The framework not only enhances computational efficiency but also accelerates convergence, achieving >98% accuracy within 8–10 federated communication rounds compared to 15–20 rounds required by standard federated ViTs. This improvement is attributed to CEQEA’s clinical-entropy-guided evolutionary optimization, which acts as an adaptive feature selector by eliminating redundant or diagnostically irrelevant tokens during local client optimization. Crucially, CEQEA’s optimization overhead remains minimal (<0.05%), ensuring that computational savings are not outweighed by additional processing costs. The efficiency gains demonstrated by the CEQEA framework represent a critical bridge to practical clinical deployment. In typical GI suites, local computing resources are often limited, and medical-grade workstations are rarely equipped with the high-end GPU arrays required by standard full-sized Vision Transformers. By achieving a significant reduction in computational complexity, our framework democratizes access to AI-driven decision support, enabling high-performance diagnostic tools to be hosted on standard hospital hardware already available within the clinical workflow. Table 6 shows the mathematical notations for the CEQEA-based FL framework.

Despite the efficacy of the proposed framework, several limitations warrant further investigation. First, the current implementation uses a fixed ViT backbone (ViT-16/B); the impact of CEQEA on larger or more lightweight transformer variants should be explored. Second, while the algorithm demonstrates robustness across heterogeneous GI datasets, its hyperparameters may require calibration when applied to medical imaging domains with substantially different visual characteristics. Third, although the framework performs well for endoscopic GI image classification, its applicability to other medical imaging modalities (e.g., radiology, pathology) and multimodal federated learning tasks remains to be validated.

## 5. Conclusions

This study introduced a federated Vision Transformer-based framework for gastrointestinal endoscopic image classification, utilizing an entropy-guided adaptive token pruning strategy to optimize performance across heterogeneous clinical environments. Our experimental evaluation demonstrates that the proposed framework maintains high diagnostic reliability—achieving mean micro-averaged accuracies of 92.33% and 90.19% on Lower and Upper GI datasets, respectively—while significantly reducing both computational complexity and communication overhead. By streamlining the federated training process, this approach enables practical, privacy-preserving collaborative model development across multi-site hospital networks. Despite these performance gains, the framework is currently constrained by the inherent variability of federated data distributions and requires further validation across more diverse, multi-national clinical cohorts. Future work will focus on integrating robust uncertainty estimation to enhance diagnostic reliability and conducting large-scale prospective clinical trials to evaluate the model’s performance within real-time procedural workflows.

## Figures and Tables

**Table 1:** Federated training and Clinical-Entropy Guided Quantum Evolutionary Algorithm (CEQEA) parameters.

Parameter	Value	Parameter	Value
ViT Model	ViT-16/B (pretrained)	Clients (*K*)	3
Learning Rate	$1\times 10^{-5}$	Batch Size	8
Local Epochs/Round	1	Communication Rounds	50
Federation	FedAvg	Participation Prob.	$\frac{2}{3}$
CEQEA Generations (*G*)	10	β (entropy scaling)	0.3
$\eta_{\mathrm{mut}}$	0.15	$\delta_{\mathrm{base}}$	0.05
α (Dirichlet)	0.5	Train/Val Split	80:20

**Table 2:** Micro- and Macro-averaged Performance Metrics for the HyperKVASIR Lower GI Dataset Across Federated Clients. Results are reported as mean ± standard deviation over 5 Monte Carlo runs with different random seeds.

Metric	Client 0	Client 1	Client 2	Aggregate Mean
Accuracy (Micro)	92.77 ± 0.21	91.72 ± 0.32	92.49 ± 0.19	92.33 ± 0.24
Precision (Micro)	92.77 ± 0.19	91.72 ± 0.28	92.49 ± 0.21	92.33 ± 0.23
Recall (Micro)	92.77 ± 0.24	91.72 ± 0.35	92.49 ± 0.23	92.33 ± 0.27
F1-score (Micro)	92.77 ± 0.20	91.72 ± 0.30	92.49 ± 0.20	92.33 ± 0.23
Specificity (Micro)	99.28 ± 0.07	99.17 ± 0.11	99.25 ± 0.08	99.23 ± 0.09
Precision (Macro)	87.22 ± 0.45	85.38 ± 0.52	86.20 ± 0.41	86.27 ± 0.46
Recall (Macro)	85.02 ± 0.48	84.20 ± 0.55	84.34 ± 0.49	84.52 ± 0.51
F1-score (Macro)	85.58 ± 0.46	84.39 ± 0.53	84.75 ± 0.45	84.91 ± 0.48

**Table 3:** Micro- and macro-averaged test performance (%) of the proposed CEQEA-based federated ViT on the HyperKVASIR Upper GI dataset. Results are reported as mean ± standard deviation over 5 Monte Carlo runs with different random seeds.

Metric	Client 0	Client 1	Client 2	Aggregate Mean
Accuracy (Micro)	90.93 ± 0.28	91.47 ± 0.22	88.17 ± 0.35	90.19 ± 0.28
Precision (Micro)	90.93 ± 0.26	91.47 ± 0.20	88.17 ± 0.31	90.19 ± 0.26
Recall (Micro)	90.93 ± 0.29	91.47 ± 0.25	88.17 ± 0.38	90.19 ± 0.31
F1-score (Micro)	90.93 ± 0.27	91.47 ± 0.21	88.17 ± 0.33	90.19 ± 0.27
Specificity (Micro)	97.86 ± 0.12	98.01 ± 0.10	97.04 ± 0.15	97.64 ± 0.12
Precision (Macro)	83.67 ± 0.55	85.41 ± 0.48	80.41 ± 0.62	83.16 ± 0.55
Recall (Macro)	84.01 ± 0.58	85.96 ± 0.51	82.27 ± 0.65	84.08 ± 0.58
F1-score (Macro)	83.47 ± 0.54	85.42 ± 0.49	81.07 ± 0.60	83.32 ± 0.54

**Table 5:** Dataset-wise computational and communication complexity comparison between standard federated ViT and the proposed CEQEA-based FL framework.

Metric	HyperKVASIR Lower GI	HyperKVASIR Lower GI	HyperKVASIR Upper GI	HyperKVASIR Upper GI
	Standard FL-ViT	CEQEA-ViT	Standard FL-ViT	CEQEA-ViT
Input image size	224×224	224×224	224×224	224×224
Total tokens per image (*N*)	197	197	197	197
Tokens retained after pruning (*K*)	197	90 (avg)	197	90 (avg)
Token pruning ratio (%)	0	54.3	0	54.3
Attention operations ($N^{2}$)	38,809	8100	38,809	8100
Attention complexity reduction (%)	0	79.1	0	79.1
Communication per FL round (%)	100	100	100	100
FL rounds to reach >98% accuracy	18	10	15	8
Total communication reduction (%)	0	44.4	0	46.7
Convergence speedup (×)	1.0	1.8	1.0	1.88
CEQEA runtime overhead (%)	0	<0.05	0	<0.05
Total training time (relative)	1.00×	0.55×	1.00×	0.53×

**Table 6:** Summary of Mathematical Notations for CEQEA-based Federated Learning Framework.

Symbol	Description	Typical Value/Dimension
*K*	Number of clients in federation	3 (experimental)
*k*	Client index	k∈{1,2,…,K}
*R*	Total communication rounds	50
*t*	Current communication round index	t∈{1,2,…,R}
$S_{t}$	Subset of active clients in round *t*	$|S_{t}|=2$
*E*	Local training epochs per round	1
*B*	Batch size for local training	8
$D_{k}$	Local endoscopic dataset at client *k*	$|D_{k}|$ varies
$n_{k}$	Number of samples at client *k*	Dataset-dependent
*n*	Total samples across all clients	$\sum_{k=1}^{K}n_{k}$
*I*	Input endoscopic image	$R^{224\times 224\times 3}$
H,W,C	Image height, width, channels	224,224,3
*p*	Patch size	16×16
$N_{p}$	Number of patches per image	$\frac{H}{p}\times \frac{W}{p}=196$
$z_{i}$	Embedding of patch *i*	$R^{768}$
$z_{\left[CLS\right]}$	Classification token embedding	$R^{768}$
*d*	Embedding dimension	768
$X_{0}$	Input sequence to transformer encoder	$R^{197\times 768}$
*L*	Number of transformer encoder layers	12
$H_{\mathrm{head}}$	Number of attention heads per layer	12
$d_{h}$	Dimension per attention head	$d/H_{\mathrm{head}}=64$
$d_{ff}$	MLP hidden dimension in transformer	4d=3072
θ	Global ViT model parameters	≈86M parameters
ϕ	Classification head parameters	Varies by number of GI classes
*C*	Number of diagnostic classes	16 (HyperKVASIR)
$H_{k}$	Clinical entropy of client *k*’s dataset	$H_{k}\in \left[0,\log_{2}C\right]$
$P_{k}$	CEQEA population size for client *k*	$P_{B}\cdot \left(1+\alpha \cdot H_{k}/\log_{2}C\right)$
$P_{B}$	Base population size	10
α	Entropy scaling factor	0.3
*G*	CEQEA generations per round	10
$m^{\left(k\right)}$	Binary pruning mask at client *k*	${\left\{0,1\right\}}^{197}$
$m^{t}$	Global aggregated mask at round *t*	${\left\{0,1\right\}}^{197}$
$K_{\mathrm{pruned}}$	Number of tokens after pruning	≈90 (avg), 120 (max)
$s_{i}$	Binary solution (individual) in CEQEA population	${\left\{0,1\right\}}^{197}$
$Q_{i}$	Quantum individual (qubit amplitudes)	$\left[\alpha_{i,1},\ldots ,\alpha_{i,N};\beta_{i,1},\ldots ,\beta_{i,N}\right]$
$\alpha_{i,j},\beta_{i,j}$	Qubit probability amplitudes	$|\alpha_{i,j}{|}^{2}+{\left|\beta_{i,j}\right|}^{2}=1$
$w_{1},w_{2}$	Fitness function weights (accuracy vs. sparsity)	0.7, 0.3
η	Mask aggregation consensus threshold	0.6
$w_{k}$	Client weight in mask aggregation	$H_{k}/\sum_{l=1}^{K}H_{l}$
$F(s_{i})$	Fitness function value for solution $s_{i}$	Equation (17)
$\Delta \theta_{ij}$	Quantum rotation angle for qubit *j* of individual *i*	Equation (18)
$\delta_{k}$	Client-adaptive quantum rotation step size	$\delta_{\mathrm{base}}\cdot \left(1+\gamma \cdot \left(1-H_{k}/H_{\max}\right)\right)$
$\delta_{\mathrm{base}}$	Base rotation step	0.05 radians
γ	Entropy scaling factor for rotation	0.3
$P_{\mathrm{mut}}^{\left(ij\right)}$	Mutation probability for qubit *j* of individual *i*	Equation (24)
$\alpha_{\mathrm{Dir}}$	Dirichlet distribution parameter for non-IID split	0.5

## Data Availability

The HyperKVASIR upper and lower GI tract datasets used in this study are publicly available at “https://www.kaggle.com/datasets/tansyab1/hyperkvasir”, accessed on 22 March 2026.

## Abbreviations

The following abbreviations are used in this manuscript:

AI	Artificial Intelligence
CEQEA	Clinical-Entropy Quantum Evolutionary Algorithm
CNN	Convolutional Neural Network
FL	Federated Learning
GI	Gastrointestinal
IID	Independent and Identically Distributed
KVASIR	Gastrointestinal endoscopy image dataset
MHSA	Multi-Head Self-Attention
ML	Machine Learning
QEA	Quantum Evolutionary Algorithm
ViT	Vision Transformer

## References

[1] Zahedi M.J., Shafieipour S., Abbasi M.M.H., Nakhaie M., Rukerd M.R.Z., Lashkarizadeh M.M., Noorbini F., Baghaei M.H., Pourjafari A., Aminian E. (2022). Mortality trends of gastrointestinal, liver, and pancreaticobiliary diseases: A hospital-based prospective study in the southeast of Iran. Middle East J. Dig. Dis..

[2] Yao L., Ji T., Mu H., Xu X. (2025). The global epidemiology of gastrointestinal cancers attributable to high-BMI: Findings from the Global Burden of Disease Study 2021. Front. Nutr..

[3] Siddiqui S., Khan J.A., Akram T., Alharbi M., Cha J., AlHammadi D.A. (2025). SNet: A novel convolutional neural network architecture for advanced endoscopic image classification of gastrointestinal disorders. SLAS Technol..

[4] Dosovitskiy A., Beyer L., Kolesnikov A., Weissenborn D., Zhai X., Unterthiner T., Dehghani M., Minderer M., Heigold G., Gelly S. (2020). An image is worth 16×16 words: Transformers for image recognition at scale. arXiv.

[5] Asad M., Shaukat S., Hu D., Wang Z., Javanmardi E., Nakazato J., Tsukada M. (2023). Limitations and future aspects of communication costs in federated learning: A survey. Sensors.

[6] Borgli H., Thambawita V., Smedsrud P.H., Hicks S., Jha D., Eskeland S.L., Randel K.R., Pogorelov K., Lux M., Nguyen D.T.D. (2020). HyperKvasir, a comprehensive multi-class image and video dataset for gastrointestinal endoscopy. Sci. Data.

[7] Khan M.A., Sahar N., Khan W.Z., Alhaisoni M., Tariq U., Zayyan M.H., Kim Y.J., Chang B. (2022). GestroNet: A framework of saliency estimation and optimal deep learning features based gastrointestinal diseases detection and classification. Diagnostics.

[8] Jha D., Sharma V., Dasu N., Tomar N.K., Hicks S., Bhuyan M.K., Das P.K., Riegler M.A., Halvorsen P., Bagci U. (2023). Gastrovision: A multi-class endoscopy image dataset for computer aided gastrointestinal disease detection. Workshop on Machine Learning for Multimodal Healthcare Data.

[9] Sedighipour Chafjiri F. (2023). Anatomical Classification of the Gastrointestinal Tract Using Ensemble Transfer Learning. Ph.D. Thesis.

[10] Chen H., Liu S., Miao Y., Yang X., Li T., Wu C., Li Z., Guo Y., Yu S., Chen G. (2025). Constructing and validating vision transformer-based assisted detection models for atrophic gastritis: A retrospective study. Sci. Prog..

[11] Ayan E. (2024). Classification of Gastrointestinal Diseases in Endoscopic Images: Comparative Analysis of Convolutional Neural Networks and Vision Transformers. J. Inst. Sci. Technol..

[12] Tanwar V., Sharma B., Yadav D.P., Mehbodniya A. (2025). Hybrid deep learning framework based on EfficientViT for classification of gastrointestinal diseases. Sci. Rep..

[13] Tang S., Yu X., Cheang C.F., Liang Y., Zhao P., Yu H.H., Choi I.C. (2023). Transformer-based multi-task learning for classification and segmentation of gastrointestinal tract endoscopic images. Comput. Biol. Med..

[14] Chou C.K., Lee K.H., Karmakar R., Mukundan A., Chen T.H., Kumar A., Gutema D., Yang P.C., Huang C.W., Wang H.C. (2025). Integrating AI with advanced hyperspectral imaging for enhanced classification of selected gastrointestinal diseases. Bioengineering.

[15] Alkhunaizi N., Almalik F., Al-Refai R., Naseer M., Nandakumar K. (2024). Probing the Efficacy of Federated Parameter-Efficient Fine-Tuning of Vision Transformers for Medical Image Classification. International Conference on Medical Image Computing and Computer-Assisted Intervention.

[16] Darzi E., Shen Y., Ou Y., Sijtsema N.M., van Ooijen P.M. (2024). Tackling heterogeneity in medical federated learning via aligning vision transformers. Artif. Intell. Med..

[17] Kirchner M., Jenke A.C., Bodenstedt S., Kolbinger F.R., Saldanha O.L., Kather J.N., Wagner M., Speidel S. (2025). Federated endovit: Pretraining vision transformers via federated learning on endoscopic image collections. arXiv.

[18] Wang C.-Y., Nguyen H.-T., Fan W.-S., Lue J.-H., Saenprasarn P., Chen M.-M., Huang S.-Y., Lin F.-C., Wang H.-C. (2024). Glaucoma detection through a novel hyperspectral imaging band selection and vision transformer integration. Diagnostics.

[19] Rao Y., Zhao W., Liu B., Lu J., Zhou J., Hsieh C.J. (2021). Dynamicvit: Efficient vision transformers with dynamic token sparsification. Adv. Neural Inf. Process. Syst..

[20] Liu X., Peng H., Zheng N., Yang Y., Hu H., Yuan Y. Efficientvit: Memory efficient vision transformer with cascaded group attention. Proceedings of the IEEE/CVF Conference on Computer Vision and Pattern Recognition.

[21] Wang Y., Huang R., Song S., Huang Z., Huang G. (2021). Not all images are worth 16x16 words: Dynamic transformers for efficient image recognition. Adv. Neural Inf. Process. Syst..

[22] Li Q., Diao Y., Chen Q., He B. Federated learning on non-iid data silos: An experimental study. Proceedings of the 2022 IEEE 38th International Conference on Data Engineering (ICDE).

[23] Archetti A., Lomurno E., Lattari F., Martin A., Matteucci M. Heterogeneous datasets for federated survival analysis simulation. Proceedings of the Companion of the 2023 ACM/SPEC International Conference on Performance Engineering.

[24] Zhao Y., Li M., Lai L., Suda N., Civin D., Chandra V. (2018). Federated learning with non-iid data. arXiv.

[25] McMahan B., Moore E., Ramage D., Hampson S., y Arcas B.A. Communication-efficient learning of deep networks from decentralized data. Proceedings of the 20th International Conference on Artificial Intelligence and Statistics.

[26] Tao J., Gao Z., Guo Z. (2022). Training vision transformers in federated learning with limited edge-device resources. Electronics.

[27] Ramamurthy K., George T.T., Shah Y., Sasidhar P. (2022). A novel multi-feature fusion method for classification of gastrointestinal diseases using endoscopy images. Diagnostics.

[28] Attallah O., Aslan M.F., Sabanci K. (2025). EndoNet: A Multiscale Deep Learning Framework for Multiple Gastrointestinal Disease Classification via Endoscopic Images. Diagnostics.

[29] Sarsengeldin M., Imatayeva S., Abeuov N., Naukhanov M., Erdogan A.S., Jha D., Bagci U. Gastrointestinal Disease Diagnosis with Hybrid Model of Capsules and CNNs. Proceedings of the 2023 IEEE International Conference on Electro Information Technology (eIT).

[30] Yue G., Wei P., Liu Y., Luo Y., Du J., Wang T. (2023). Automated endoscopic image classification via deep neural network with class imbalance loss. IEEE Trans. Instrum. Meas..

[31] Wang W., Yang X., Tang J. (2023). Vision transformer with hybrid shifted windows for gastrointestinal endoscopy image classification. IEEE Trans. Circuits Syst. Video Technol..

[32] Tabassum S., Ahamed M.F., Kibria H.B., Nahiduzzaman M., Haider J., Chowdhury M.E., Islam M.T. (2025). GastroViT: A Vision Transformer Based Ensemble Learning Approach for Gastrointestinal Disease Classification with Grad CAM & SHAP Visualization. arXiv.

[33] Devkota A., Amireskandari A., Palko J., Thakkar S., Adjeroh D., Jiang X., Bhattarai B., Gyawali P.K. (2025). Federated Foundation Model for GI Endoscopy Images. arXiv.

